# Synthesis of alkynyl-substituted camphor derivatives and their use in the preparation of paclitaxel-related compounds

**DOI:** 10.3762/bjoc.13.122

**Published:** 2017-06-26

**Authors:** M Fernanda N N Carvalho, Rudolf Herrmann, Gabriele Wagner

**Affiliations:** 1 CQE, Instituto Superior Técnico, Universidade de Lisboa, Av. Rovisco Pais, P-1049-001 Lisbon, Portugal; 2 Institute of Physics, University of Augsburg, Universitätsstr. 1, D-86135 Augsburg, Germany; 3 Department of Natural Sciences, University of Chester, Thornton Science Park, Pool Lane, Ince, Chester, CH2 4NU, United Kingdom

**Keywords:** alkynes, camphor derivatives, catalysis, cycloisomerisation, platinum

## Abstract

Compounds containing two alkyne groups in close vicinity at the rigid skeleton of camphorsulfonamide show unique reactivities when treated with electrophiles or catalytic amounts of platinum(II). The formed product structures depend not only on the reagents used but also on the substituents attached to the triple bonds. Cycloisomerisations with perfect atom economy lead to polycyclic heterocycles that resemble to some extent the AB ring system of paclitaxel. Herein, we present practical synthetic methods for the selective synthesis of precursor dialkynes bearing different substituents (alkyl, aryl) at the triple bonds, based on ketals or an imine as protecting groups. We show for isomeric dialkynes that the reaction cascade induced by Pt(II) includes ring annulation, sulphur reduction, and ring enlargement. One isomeric dialkyne additionally allows for the isolation of a pentacyclic compound lacking the ring enlargement step, which we have proposed as a potential intermediate in the catalytic cycle.

## Introduction

Enantiomerically pure raw materials, available in a sustainable manner from the natural “chiral pool” [1], offer a convenient entrance for the chemical synthesis of other chiral compounds, e.g., rare natural products and their analogues [2], or chiral catalysts [3–4]. A prominent example for such a “chiral pool” starting material is camphor. Both its substituents and its bicyclic skeleton can easily be modified and adapted to the purpose at hand, e.g., natural product synthesis [5]. The Wagner–Meerwein and Nametkin-type rearrangements are the most common reaction patterns [6] and the addition of organometallic reagents to the camphor carbonyl group allows for selective introduction of additional substituents and functional groups (e.g., as in [7]). Camphor was the source of chirality in Holton’s taxol synthesis [8] and other approaches to the taxane group of compounds [9–11].

**Scheme 1:**
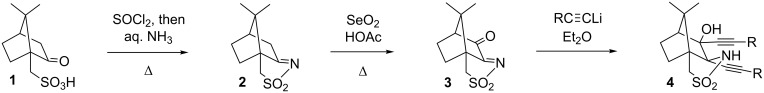
Synthesis of 3-oxo-camphorsulfonylimine (**3**) [13,15] and its bis-alkynyl derivatives **4** from camphor-10-sulfonic acid (**1**).

From cheap camphor-10-sulfonic acid (**1**), a cyclic sulfonimide **2** can easily be obtained which is readily converted into useful auxiliaries [12], or oxidized to the oxoimide **3** (Scheme 1) [13–15]. This versatile intermediate can be reduced to provide a chelating ligand for chiral catalysis [16], or oxidised to oxaziridines used as efficient chiral oxidising reagents [13,17–19].

**Scheme 2:**
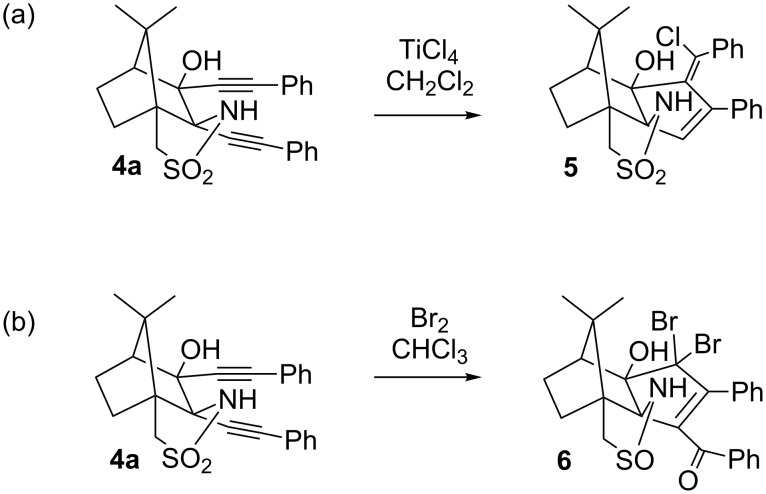
Reactions of bis-alkynyl camphor derivative **4a** with TiCl_4_ and with Br_2_, respectively.

The reaction of the oxoimide **3** with two equivalents of the lithium salt of a terminal alkyne leads to compounds **4** where two alkynyl substituents, a sulfonamide and a hydroxy group are found in vicinal positions (Scheme 1) [20]. A hydroxy group neighbouring an alkynyl substituent, under treatment with acids, normally leads to Rupe and Meyer–Schuster rearrangements, forming unsaturated carbonyl compounds. This was indeed observed in camphor-derived bicyclic alcohols containing a single ethinyl group [21–22], occasionally accompanied by a Wagner–Meerwein rearrangement [23]. However, no such products were found with any of the diynes **4**. Our first attempts to employ **4a** as a ligand with Ti(IV) resulted, somewhat surprisingly, in the addition of HCl under simultaneous annulation (three-carbon expansion [24–25]) of a carbocyclic five-membered ring to the 2,3-position of the bicyclic camphor-derived moiety (Scheme 2a) [20]. Reactions of **4a** with halogens (e.g., bromine) or acids were even more puzzling. In addition to the annulation, an unprecedented formation of a ketone accompanied by the reduction of sulphur took place, to give a cyclic sulfinamide **6** (Scheme 2b) [26]. In this case, the mechanism of the reaction proceeded through cationic intermediates as evidenced by in situ NMR spectroscopy.

**Scheme 3:**
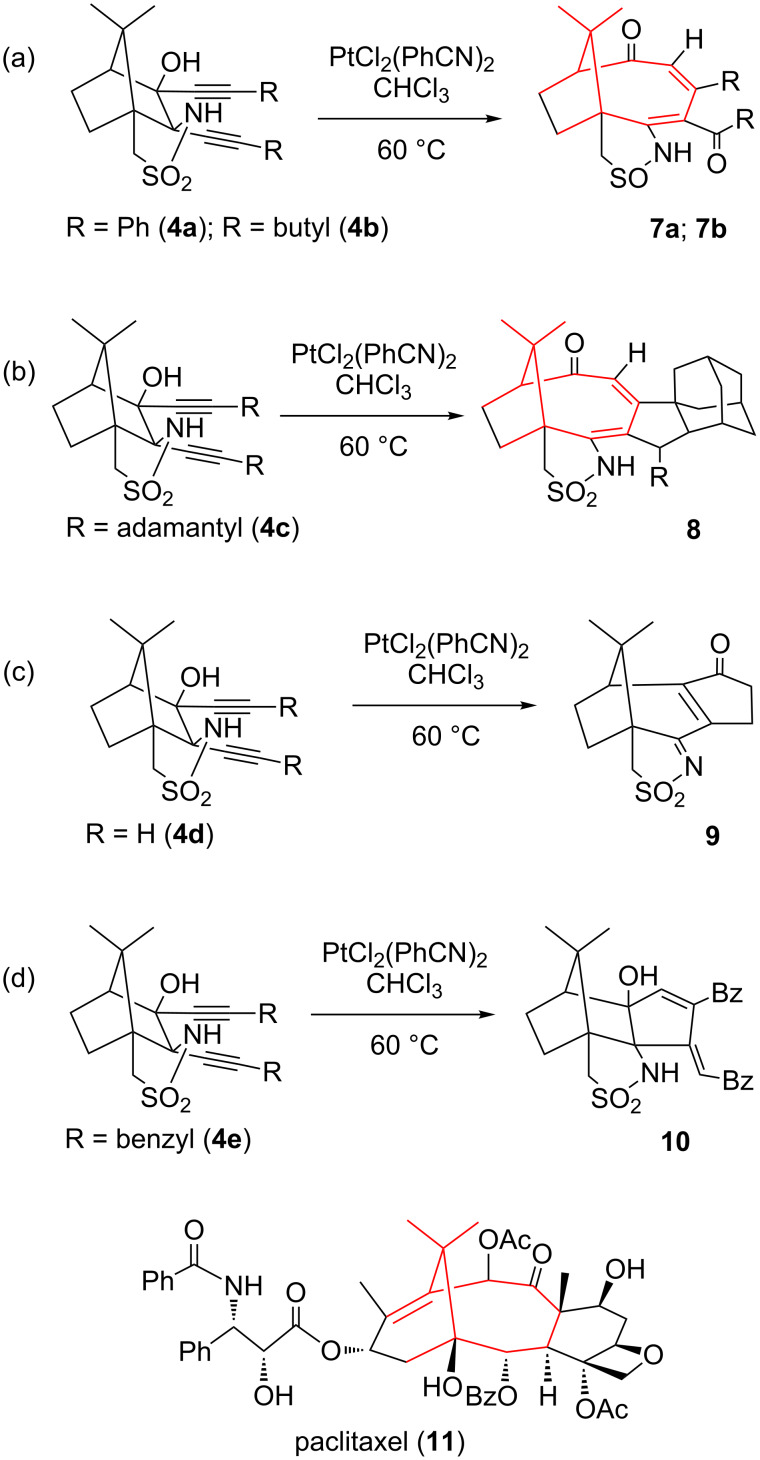
Reactions of bis-alkynylcamphor derivatives **4a–e** with catalytic amounts of PtCl_2_(PhCN)_2_.

Catalysis by Pt(II) can drive the reaction even further: besides annulation and sulphur reduction, one finds a cleavage of the C–C bond between the atoms bearing the OH and NH groups (ring enlargement). The result is an isomerisation of **4a** and **4b** to form tricyclic compounds **7** containing a nine-membered carbocyclic ring (Scheme 3a) [27]. Isomerisations are the best examples for a perfect “atom economy” [28–30] since all atoms of the starting material are found in the product, and thus fulfil an important requirement of “green chemistry” [31].

However, a different Pt(II)-catalysed reaction cascade was observed for **4c**, with adamantyl groups at the alkynes. Here, the annulation step is followed by a C–H bond-activation process, to establish an additional bond to one of the adamantyl groups (**8** in Scheme 3b) [32]. The simplest diyne **4d** with R = H, in contrast, gave a ring enlargement from six to seven members together with a 1,2-oxygen shift instead (**9** in Scheme 3c) [33]. This clearly demonstrates that the substituents at the triple bonds have a decisive influence on the outcome of the catalytic reaction. This was also confirmed by the reaction of **4e** (R = benzyl): in addition to the expected product **7** in analogy to that of **4b**, there was also a considerable amount of a reduced species **10** lacking sulphur reduction and ring enlargement, with structural similarity to the simple product from the Ti(IV) reaction with **4a**. The reducing agent is Pt(II), which is oxidised to Pt(III) during the reaction (Scheme 3d) [34].

Scheme 3 also depicts paclitaxel (taxol, **11**), an important anticancer drug, as there are some similarities (shown in red in Scheme 3) but also differences to our compounds obtained by Pt(II) catalysis from, e.g., **4a**. The eye-catching dimethylmethylene bridge over the largest carbocyclic ring is of course the most striking similarity, although this ring is in our compounds one-carbon unit smaller (9 vs 10 members in taxol) and lacks the oxygen substituent bearing the amino acid side group. In both compounds, we find a keto group in the largest ring. Instead of the six-membered ring annulation in taxol, there are the substituents of the original triple bonds in our compounds, precisely in the same positions. In addition, where taxol has the bridgehead hydroxy and its neighbouring benzoate groups, we find the heterocyclic reduced isothiazole ring in the product of the Pt(II) catalysis. Other camphor derivatives prepared as entrance to taxoid compounds have a carbocyclic ring at this place in the precursor [10] and a nine-membered ring with a keto group after oxidative bond cleavage [11]. The similarities between our materials and taxol do, of course, not mean that similar biological activity is necessarily involved, but it might be worthwhile investigating.

Since the results of the Pt(II) catalysis depend on the nature of the substituents at the alkyne groups, it would be of interest to explore the course of the reaction when the two substituents are different. In this article, we develop reasonable synthetic procedures for the starting diynes, and present the first result of a catalytic Pt(II) reaction of such mixed substituted compounds.

## Results and Discussion

For the preparation of the diynes **4**, a 2:1 ratio (or slightly larger for complete reaction) of the lithium salt of a terminal alkyne and of the oxoimide **3** is applied. The ratio should, however, not be increased too much. For instance, with benzylacetylene, the expected diyne **4e** (Scheme 3d) is obtained, with only traces of monosubstituted compounds. However, with a 3:1 ratio, the main product is formed by reaction of only one equivalent of lithium salt with the C=N double bond, leaving the carbonyl group intact. In addition, the formed propargyl group is isomerized to an allene moiety, obviously due to the excess of the strongly basic lithium salt [35]. This unexpected monosubstitution remains unexplained and is not applicable as basis for a general selective synthesis of monoalkynylcamphor derivatives. Alkynes can also be cleaved from the bis-alkynyl compounds **4** by reaction with CuCl, but again, there are selectivity issues that prevent a general application [36]. We therefore set out to explore whether some selectivity is observed when lithium salts of terminal alkynes are reacted with the oxoimide **3** in a 1:1 ratio (Table 1). As alkyne precursors, phenylacetylene, 1-heptyne, 1-ethynyladamantane, and 1-ethynyl-1-methoxycyclohexane were used. In all cases, mixtures of **12** and **13**, together with starting oxoimide **3** and the bis-alkynyl product **4** were obtained. The very slow addition of the acetylide and dilute oxoimide solutions slightly improved the yields of the mono-adducts **12** and **13**, but the formation of the bis-adducts could not be fully suppressed. With increasing bulkiness of the alkyne substituent (R = adamantyl, methoxycyclohexyl) the reaction tends to become more selective towards mono-addition, but less selective with respect to the alkylation site, and the 2-alkynyl and 3-alkynyl products are formed in similar amounts. Alkynes with small substituents (e.g., heptyne or phenylacetylene) preferentially attack at the C=N bond of the sulfonylimine, suggesting that the carbon atom of the C=N is more electrophilic than that of the C=O bond. However, with bulkier alkynes (e.g., 1-adamantylacetylene or 1-methoxy-1-ethynylcyclohexane), an attack at the carbonyl group becomes more pronounced. Presumably, the C=O carbon atom in 3-oxo-camphorsulfonylimine is sterically more accessible, whereas the sulfonylimine C=N carbon is more electrophilic. Steric and electronic properties thus counteract and the overall selectivity of the reaction depends on a balance between both factors.

**Table 1:** Selectivity in the reaction of oxoimide **3** with alkynyllithium compounds.

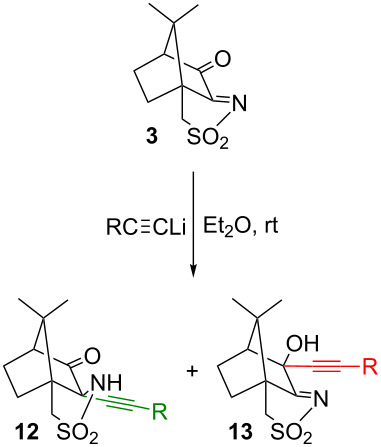			

Entry	R	**12**:**13**	Yield **12** + **13**

1	phenyl	80:20	42%
2	*n*-pentyl	90:10	35%
3	1-adamantyl	70:30	88%
4	1-methoxycyclohexyl	50:50	67%

Whilst chromatographic separation of the mixture of the monoadducts **12** + **13** from the starting material **3** and the bis-adduct **4** is straightforward, the isolation of pure **12** and **13** requires careful control of the chromatography conditions (SiO_2_, CHCl_3_/diethyl ether gradient 0 to 10%), and has been used on a small scale for analytical purposes only. The ratios **12**:**13** given in Table 1 were determined by integration of the signals of the methyl groups in the ^1^H NMR. The ratios in the crude product and after isolation of the mixture of **12** and **13** by chromatography were found consistent.

In the IR spectra, compounds **12** show a typical NH stretching vibration at approximately 3217 cm^−1^, and a C=O stretch at 1764 cm^−1^. Compounds **13**, in contrast, can be recognised by their OH stretching vibration at 3444 cm^−1^ and the C=N stretch at 1653 cm^−1^. Both compounds display a C≡C stretch at very similar wavenumbers around 2227 cm^−1^, and asymmetric and symmetric SO_2_ stretching vibrations. These are found at about 1314 and 1143 cm^−1^ in compounds **12**, and at slightly higher wavenumbers of approx. 1330 and 1160 cm^−1^ in **13**. In the ^1^H NMR spectra, the signals of the geminal methyl groups come with a chemical shift difference of about 0.2 ppm in **12**, but almost coincide to form one signal in compounds **13**. The opposite holds true for the diastereotopic protons of the CH_2_SO_2_ moiety, which nearly coincide in **12**, but come as two doublets spaced about 0.2 ppm apart in **13**. Compounds **12** display a singlet near 5 ppm for the SO_2_NH proton, whereas spectra of **13** show a singlet for the OH group at about 3.2 ppm. Similarly, the ^13^C NMR signals of the methyl groups nearly coincide in **13** but are approximately 2 ppm apart in **12**. Compounds **12** show a signal for the C=O near 206 ppm and one for the sulfonamide carbon at about 65 ppm. In compounds **13**, there is a signal for the C=N around 194 ppm and one for the tertiary alcohol carbon near 73 ppm. All other signals of the camphor framework and the alkynyl substituent come at relatively similar values and do not allow distinguishing between **12** and **13** reliably.

**Scheme 4:**
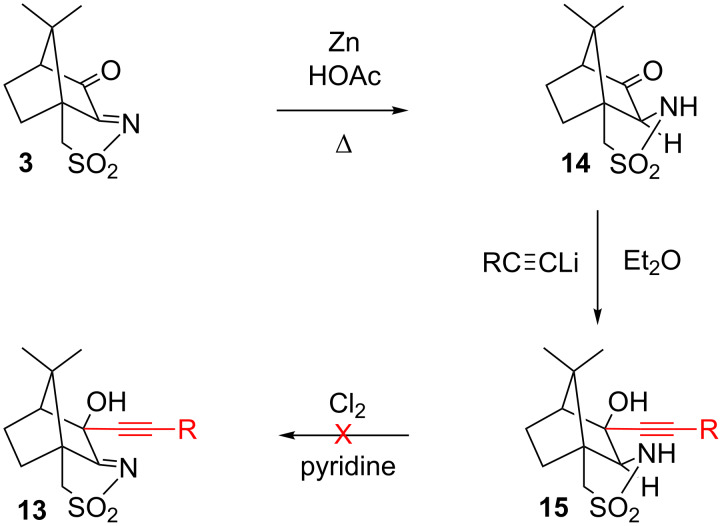
Attempted selective synthesis of 3-alkynyl derivatives via sulfonylimine reduction of oxoimide **3**.

In view of the above results, it was clear that a more selective method was needed that generally allows for mono-alkynylation and for differentiation between the carbonyl group and the sulfonylimine. One way to achieve this could be in the chemoselective reduction of the C=N group of oxoimide **3**, addition of the alkynyl moiety to the carbonyl group, and re-oxidation of the sultam to the sulfonimide (see Scheme 4). There are a number of reductions of oxoimide **3** reported in the literature, using a variety of reducing agents. All of them lead either to complete or to unselective reduction of the C=N and C=O bond. Thus, LiAlH_4_ reduces the C=O and C=N group of **3**, and 3-*exo*-hydroxycamphorsultam is obtained in good yields [16]. Under Meerwein–Ponndorf–Verley conditions (Al(OiPr)_3_/iPrOH), a mixture of the 3-hydroxyimine, 3-oxocamphorsultam and 3-*exo*-hydroxycamphorsultam is produced. Prolonged reaction over several weeks eventually leads to 3-*exo*-hydroxycamphorsultam as the sole product.

We now found that the sulfonylimine of **3** could be reduced selectively to the sultam **14** in the presence of Zn/HOAc, as shown in Scheme 4. The product shows an IR stretching vibration at 1760 cm^−1^ and a ^13^C NMR signal at 209.7 ppm for the unreacted C=O group. The presence of the sultam can be deduced from the NH stretching vibration at 3180 cm^−1^, a ^13^C signal of the sulfonamide carbon at 65.0 ppm and a ^1^H NMR signal for the NH at 5.57 ppm. These data are very similar to those of compound **12** where C=O and sulfonamide coexist. In contrast to **12**, the ^1^H NMR signal of the NH in **14** comes as a doublet, due to coupling with the adjacent CH proton at 3.58 ppm. The latter proton also appears as a doublet. The introduction of an alkynyl substituent into the 3-position does not pose any problem and can be achieved by reaction with PhC≡CLi under standard conditions, to produce **15** in high yield. Two equivalents of the acetylide are required because the first one deprotonates the nitrogen of the sultam before the second equivalent undergoes the desired nucleophilic addition to the carbonyl group. All attempts to re-oxidise the sultam **15** to the sulfonimide **13**, using Cl_2_/pyridine [37] or *N*-*tert*-butylphenylsulfinimidoyl chloride/DBU [38] under literature conditions, were unsuccessful due to the sensitivity of the alkyne to oxidising conditions.

**Scheme 5:**
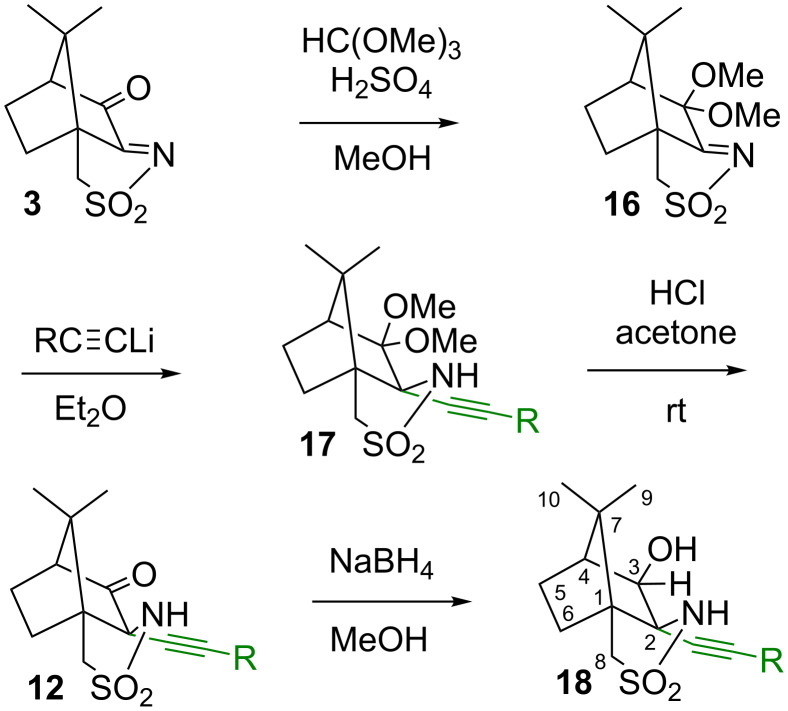
Selective synthesis of 2-alkynyl derivatives by protection of the 3-oxo group as an acetal.

**Scheme 6:**
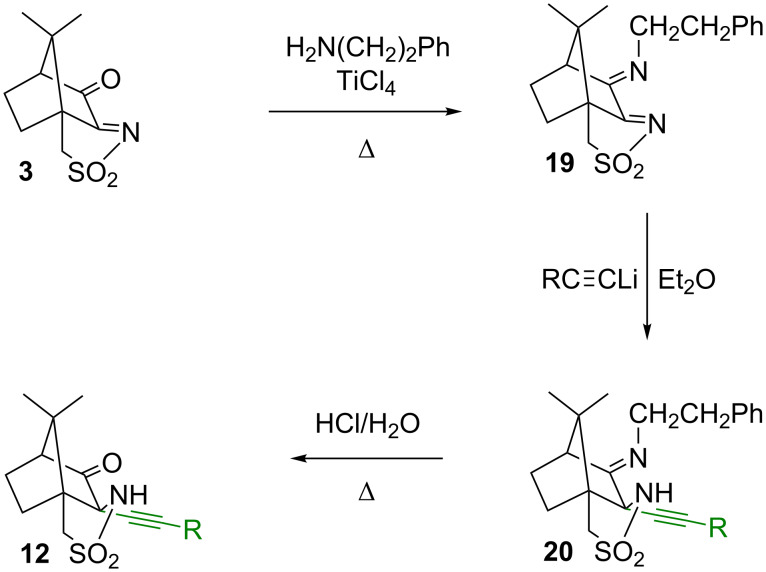
Selective synthesis of 2-alkynyl derivatives by protection of the 3-oxo group as an imine.

Our next strategy was to modify the carbonyl group in the 3-position and introduce the alkyne at the sulfonamide side, as shown in Scheme 5 and Scheme 6. The carbonyl group in **3** can be selectively protected as an acetal by reaction with orthoformates at room temperature in the presence of an acid. Thus, 3,3-dimethoxycamphersulfonylimine **16** and 3,3-diethoxycamphersulfonylimine **16’** were prepared [18,39]. The subsequent reaction of these acetals with one equivalent of lithium phenylacetylide or 1-heptynyllithium under conditions described above for the synthesis of **12** and **13** introduced the alkyne into the 2-position of the camphor skeleton, to provide the sultams **17**. The removal of the acetal-protecting group occurred under comparatively mild conditions by stirring a mixture of **17** with acetone and conc. HCl. Acetone as solvent was best as it allows for *trans*-acetalisation to acetone dimethylacetal and **12** rather than hydrolysis to methanol and **12**. Overall, yields are higher throughout when the methyl acetal is used. To obtain the camphor-derived hydroxysultam **18** bearing an alkyne substituent in 2-position rather than in 3-position, **12** was reduced with NaBH_4_ under standard conditions. Compound **18** was prepared for comparison of the analytical data with that of compound **15**. Although the IR spectra look fairly similar, the two isomers can be distinguished from their NMR spectra, in particular the ^1^H and ^13^C signals of the atoms in 2 and 3 position. Thus, the proton in the 3-position in **18** comes at higher chemical shift than the one in the 2-position in **15** (4.31 ppm vs 3.67 ppm). Likewise, the signals of carbons 2 and 3 are further downfield, when hydrogen is attached to them. In addition, carbon 4 is affected and appears at higher chemical shift in compound **15**, where the alkynyl substituent is nearby.

As an alternative to the introduction of an acetal, an imine was tested for its suitability as a protecting group for the carbonyl moiety, as shown in Scheme 6. 3-Oxocamphorsulfonylimine **3** was converted into the imine **19** by reaction with 2-phenylethylamine in the presence of TiCl_4_ [40]. The reaction of **19** with one equivalent of the lithium phenylacetylide under the conditions described above for the synthesis of **12** and **13** provided the sultam **20** selectively, with exclusive introduction of the alkyne into the 2-position at the camphor skeleton [41]. When two equivalents of the acetylide are used, the reaction still produces **20** only, and no reaction at the 3-position in the imine was observed. The high selectivity of this reaction can be explained by the large difference between the electron-deficient sulfonylimine and the electron-rich imine. The sulfonylimine carbon atom is significantly more electrophilic and thus more prone to attack by the acetylide. In the case of 3-oxocamphorsulfonylimine, this difference between the reactive functional groups (C=O vs C=N-SO_2_R) was much less pronounced, leading to poor selectivity. The general ^1^H and ^13^C signal pattern of the camphor skeleton in compound **20** is fairly similar to the one found for compounds **12**, indicating that the alkyne has indeed been introduced at the sulfonamide side. Also the ^13^C signal at 175.8 ppm shows clearly the presence of the imino group in 3-position. For a sulfonimide, a signal at higher ppm values (approx. 190 ppm) would have been expected. The removal of the imine-protecting group turned out to be somewhat difficult and could be performed only under relatively harsh acidic conditions using aqueous HCl under reflux to provide **12** in moderate yield. Overall, the protection of the carbonyl group as an acetal appears more convenient than as an imine.

**Scheme 7:**
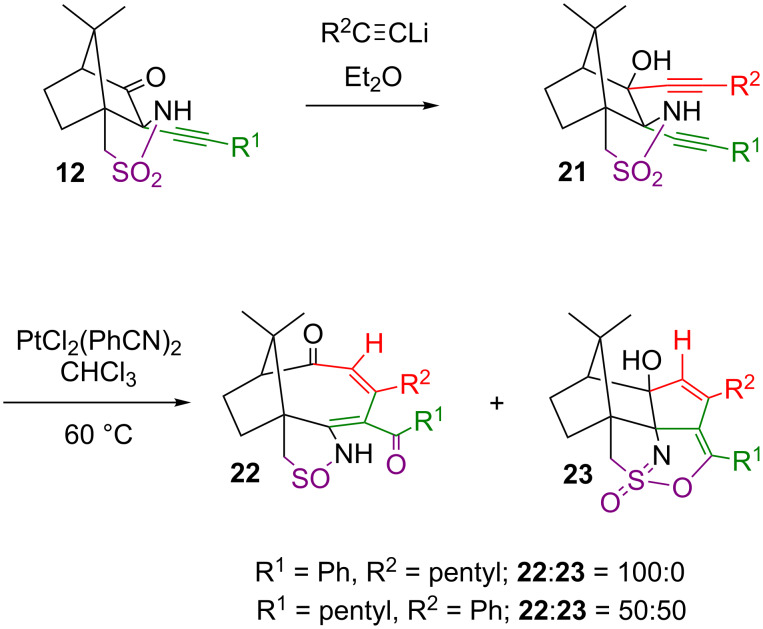
Synthesis of the bis-alkynyl derivatives bearing different alkyne substituents and their platinum-catalysed cycloisomerisation. Compounds **21a** and **22a**: R^1^ = Ph, R^2^ = pentyl; compounds **21b**, **22b** and **23**: R^1^ = pentyl, R^2^ = Ph.

Starting from the 2-alkynyl-3-oxo compound **12a**, the mixed bis-alkynyl compound **21a** was prepared by reaction with 1-heptynyllithium, and accordingly, **21b** was obtained from reaction of **12b** with PhC≡CLi (Scheme 7) [41–42]. Two equivalents of the alkynyllithium compound are necessary because the first one is required for the deprotonation of the relatively acidic proton at the sulfonamide nitrogen.

Compounds **21a** and **21b** were then reacted with 5 mol % PtCl_2_(PhCN)_2_ at 60 °C in CHCl_3_ [42]. For ^1^H NMR monitoring, the same conditions were applied, but CDCl_3_ was used as solvent. Within 10 hours, compound **21a** converts cleanly into product **22a** without the observation of intermediates or side products. Under microwave irradiation, the reaction is complete within 30 min at 80 °C, without impairing the selectivity. The structure of **22a** is analogous to the one observed previously with the bis-phenylalkynyl compound [27] as a starting material. Cyclisation of the alkynes and a three-carbon ring enlargement lead in a single step to a rare bicyclic carbon framework that bears some similarity to that of the anticancer drug paclitaxel. Remarkably, the sulfonamide group was reduced to a sulfinamide in the course of the reaction, and one of the former alkynyl carbons was oxidised to a ketone. The presence of the sulfinamide can be deduced from the fragmentation pattern in the mass spectrum where the loss of SO can be seen which is further supported by the IR spectrum. Only one S=O vibration can be identified at 1098 cm^−1^, in a similar position as the S=O vibrations in DMSO (1050 cm^−1^) or *tert*-butylsulfinamide (1032 cm^−1^) [43]. The strong band at about 1330 cm^−1^, typical for the asymmetric S=O stretch in sulfonamides and sulfones, is absent in **22a**. The IR spectrum also shows the presence of two carbonyl groups at 1680 and 1611 cm^−1^, and these are further confirmed in the ^13^C NMR spectrum (signals at 198.3 and 210.8 ppm). The NH of the sulfinamide seems to be strongly involved in hydrogen bonding with the carbonyl group nearby, as evidenced from the NH stretching vibration in the IR at 3110 cm^−1^ and the ^1^H NMR signal at an unusually high chemical shift of 11.97 ppm. The ring-expanded carbon skeleton has been corroborated from ^1^H/^13^C NMR together with 2D experiments, which allowed for a complete and unequivocal assignment of all signals.

Upon reaction with catalytic amounts of PtCl_2_(PhCN)_2_, compound **21b** converts into two products, apparently in a parallel reaction, and these were separated by chromatography. One product is the expected **22b**, whose structure is analogous to **22a** described above. The other one, **23**, has undergone alkyne cyclisation but the ring expansion has not yet taken place. The sulfonamide reduction and formation of the ketone are just about to occur, as the oxygen atom involved is on its way of being transferred from the sulphur onto the carbon atom. The structure of **23** was established by two-dimensional ^1^H/^13^C NMR experiments, and by comparison with relatively similar compounds obtained in the reaction of the bis(phenylalkynyl)-derivative of **4a** with triflic acid [26], or with K[ReO_4_] [27]. The polycyclic carbon skeleton in these is identical, but the latter compounds are protonated at the sulfonimide nitrogen and form the triflate or perrhenate salts. The newly formed five-membered ring in **23** is evident from the ^13^C NMR signals at 84.0, 90.0, 138.5, 136.0 and 125.5 ppm, and a ^1^H signal at 5.80 ppm. The enolate carbon is detected at 151.4 ppm in ^13^C NMR. Neither NMR nor IR show any evidence for carbonyl groups, but there is a medium intense C=C stretching vibration at 1653 cm^−1^ and two strong bands at 1324 cm^−1^ and 1059 cm^−1^ for the asymmetric and symmetric stretches of the O=S=N moiety. Compared to the SO_2_ moiety in sulfonamides (e.g., **21b** with 1330 cm^−1^ and 1128 cm^−1^) the symmetric stretch is at unusually low wavenumbers.

**Scheme 8:**
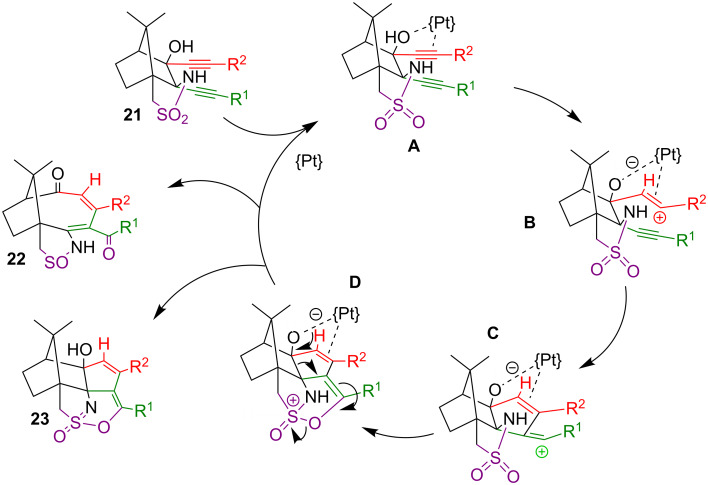
Proposed mechanism of the platinum-catalysed cycloisomerisation.

The formation of products **22** and **23** can be explained from the proposed reaction mechanism, shown in Scheme 8. The attack of the catalyst at the OH and possibly at the alkyne in the immediate neighbourhood could be inferred from the fact that the ^1^H signal of the OH of **21** broadens significantly upon addition of PtCl_2_(PhCN)_2_. Concomitantly, a small amount of uncoordinated PhCN is observed in the ^1^H NMR spectrum. The coordinated Pt centre, as a Lewis acid, promotes the release of the OH proton from **A** and its transfer onto one of the alkyne carbon atoms to form intermediate **B**. The resulting vinylic carbocation undergoes an electrophilic attack at the neighbouring alkyne, which in turn reacts with the sulfonamide oxygen nearby. Both steps are strongly facilitated by the geometry changes when the linear alkyne moieties convert into bent alkene ones, as the reactive centre is literally pushed into the functional group it next reacts with. The intermediate **D** can be regarded as the common precursor for the parallel formation of the observed products, **23** by dissociation from the Pt catalyst and proton migration from the sulfonamide to the alcoholate, and **22** by a cascade of electron movements as indicated by the arrows in **D**.

## Conclusion

Several methods for the synthesis of camphor-derived dialkynes having two different alkynyl substituents in close vicinity to each other and to a sulfonamide group were developed. Ketals turned out to be most efficient for the protection of carbonyl groups, leading to pure dialkynes with a well-defined substitution pattern. The reactivity of a pair of isomers containing a phenyl and a pentyl group attached to the triple bonds towards cycloisomerisation induced by Pt(II) catalysis was studied. The expected annulation–sulphur reduction–ring enlargement cascade leading to a product resembling paclitaxel to some extent was found in both cases. However, one of the isomers yielded a second product lacking the ring-enlargement step and containing an additional sulphur–oxygen–carbon linkage. The platinum complex of this compound was postulated before as an intermediate in the sulphur-reduction step of the cascade reaction, and the isolated product thus supports our mechanistic considerations. Platinum(II) catalysis applied to camphor-derived dialkynes with two different substituents can thus not only give valuable insight in the mechanism of such cycloisomerisations and help to clarify the role of the substituents, but also yields a novel type of taxoid compounds of complex polycyclic structures with potential biological effects.

## Supporting Information

File 1 Experimental procedures and copies of ^1^H and ^13^C NMR spectra of compounds **12a**, **12b**, **13a**, **17a**, **17b**, **18**, **19**, **20**, **21a**, **21b**, **22a**, **22b** and **23**.
