# Supplementary material for: Synthesis of alkynyl-substituted camphor derivatives and their use in the preparation of paclitaxel-related compounds
Source: Beilstein J Org Chem. 2017 Jun 26;13:1230–8. doi: 10.3762/bjoc.13.122 (PMC5496576; doi:10.3762/bjoc.13.122)

Supporting Information

for

Synthesis of alkynyl-substituted camphor derivatives and their use in the preparation of paclitaxel-related compounds

M. Fernanda N. N. Carvalho[1], Rudolf Herrmann[2] and Gabriele Wagner[3,§*]

Address: [1]CQE, Instituto Superior Técnico, Universidade de Lisboa, Av. Rovisco Pais, P-1049-001 Lisbon, Portugal, [2]Institute of Physics, University of Augsburg, Universitätsstr. 1, D-86135 Augsburg, Germany and [3]Department of Natural Sciences, University of Chester, Thornton Science Park, Pool Lane, Ince, Chester, CH2 4NU, United Kingdom

Email Gabriele Wagner - g.wagner@chester.ac.uk

*Corresponding author

[§]Tel.: +44 (0) 1244 512427

Experimental procedures and copies of ^{1}H and ^{13}C NMR spectra of compounds **12a**, **12b**, **13a**, **17a**, **17b**, **18**, **19**, **20**, **21a**, **21b**, **22a**, **22b** and **23**

Content:

Experimental section..S2

References..S19

Copies of ^{1}H and ^{13}C NMR spectra..S20

Experimental section

Materials and instrumentation. 3-Oxo-camphorsulfonylimine (**3**) [1,2] and the 3,3-dialkoxy-camphorsulfonylimines **16** and **16'** [3] were prepared according to the literature. Reactions involving carbanions or $TiCl_4$ were carried out in an N_2 atmosphere. Syntheses under microwave irradiation were performed in an Anton Paar Monowave 400 reactor in sealed vials. C, H, N and S elemental analyses were carried out on VarioEL III CHNS and Leeman CE-440 CHN elemental analyser. Melting points were determined with a Büchi 530 apparatus in open capillaries. For TLC, Merck UV 254 SiO_2-plates have been used. Mass spectra were obtained on a Thermoquest MAT 95XL instrument. Infrared spectra (4000–400 cm^{-1}) were recorded on Perkin Elmer 2000 FTIR and Nicolet Avatar 320 FTIR instruments as KBr pellets. ^1H and ^{13}C one- and two-dimensional NMR experiments were performed on Varian UNITY 300, Varian MERCURYplus 400, Bruker AM 360, Bruker AV 500 and Bruker DRX 500 spectrometers at ambient temperature. Signals were assigned with the help of COSY, NOESY, HMQC, HSQC and HMBC spectra.

Preparation of the Monoalkyne derivatives 12 and 13 from 3-oxo-camphorsulfonyl-imine (3)

Similar as described in [4], a solution of the alkyne (2.1 mmol) in dry diethyl ether (5 mL) was cooled in an ice bath. Butyl lithium (1.6 M in hexanes, 1.25 mL, 2 mmol) was added and the reaction mixture was left at room temperature for 30 min, before it was added dropwise to a suspension of 3-oxo-camphorsulfonylimine (**3**) (460 mg, 2 mmol) in dry diethyl ether (5 mL). The reaction mixture was stirred overnight, water (5 mL) was added and the organic phase was separated. The aqueous phase was extracted twice with dichloromethane, and the combined organic phases were dried with Na_2SO_4. After chromatography on SiO_2 (eluent CH_2Cl_2/Et_2O 9:1) the mixture of the 2- and 3-substituted compounds **12** and **13** was obtained as a colourless oil (**12** and **13** elute together). From this mixture, the ratio **12**:**13** was determined by ^1H NMR spectroscopy. Compounds **12** and

13 could be separated by chromatography on SiO_2, using a $CHCl_3$/diethylether gradient 0 to 10% as eluent.

a) Reaction with phenylacetylene:

Yield is 42%, **12a**:**13a** = 80:20. Anal. Calcd for $C_{18}H_{19}NO_3S$: C, 65.65; H, 5.81; N, 4.25; S, 9.71. Found: C, 65.40; H, 5.80; N, 4.51; S, 9.68. CI-MS, *m/z*: (M is 329.421) 330 $[M + H]^+$.

(3a*S*,7a*S*)-8,8-Dimethyl-7-oxo-7a-phenylethynyl-1,4,5,6,7,7a-hexahydro-3*H*-3a,6-methano-2,1-benzisothiazole 2,2-dioxide (12a)

Colourless solid. M.p. 147-148 ^oC. TLC on SiO_2, R_f = 0.40 (eluent $CHCl_3/Et_2O$ 9:1). IR spectrum (selected bands), cm^{-1}: 3218 s ν(NH), 2226 w ν (C≡C), 1760 s ν (C=O), 1312 and 1143 s ν (SO_2). ^1H NMR spectrum (500 MHz) in $CDCl_3$, δ (ppm): 1.08 (s, 3H) and 1.26 (s, 3H)(H-9 and H-10), 1.89 (m, 1H), 2.08 (m, 2H) and 2.48 (m, 1H)(H-5 and H-6), 2.48 (m, 1H, H-4), 3.42 (d, J = 13.0 Hz, 1H) and 3.46 (d, J = 13.0 Hz, 1H)(H-8), 5.44 (s, 1H, NH), 7.30 (m, 3H) and 7.45 (m, 2H)(Ph). ^{13}C NMR spectrum (125 MHz) in $CDCl_3$, δ (ppm): 19.9 and 22.8 (C-9 and C-10), 21.9 (C-5), 29.2 (C-6), 45.4 (C-7), 49.9 (C-8), 57.4 (C-1), 58.6 (C-4), 65.5 (C-2), 84.3 and 90.0 (C≡C), 121.6, 128.5, 129.4 and 132.2 (Ph), 205.7 (C-3).

(3a*S*,7*R*)-7-Hydroxy-8,8-dimethyl-7-phenylethynyl-4,5,6,7-tetrahydro-3*H*-3a,6-methano-2,1-benzisothiazole 2,2-dioxide (13a)

Colourless solid. M.p. 160-162 ^oC. TLC on SiO_2, R_f = 0.38 (eluent CH_2Cl_2/Et_2O 9:1). IR spectrum (selected bands), cm^{-1}: 3442 s ν(OH), 2223 w ν (C≡C), 1652 s ν (C=N), 1334 and 1166 s ν (SO_2). ^1H NMR spectrum (500 MHz) in $CDCl_3$, δ (ppm): 1.15 (s, 3H) and 1.16 (s, 3H)(H-9 and H-10), 1.90 (m, 1H), 2.09 (m, 2H) and 2.32 (m, 1H)(H-5 and H-6), 2.44 (d, J = 4.6 Hz, 1H, H-4), 3.20 (d, J = 13.2 Hz, 1H) and 3.33 (d, J = 13.2 Hz, 1H)(H-8), 3.41 (s, 1H, OH), 7.37 (m, 3H) and 7.50 (m, 2H)(Ph). ^{13}C NMR spectrum (125 MHz) in $CDCl_3$, δ (ppm): 21.0 and 21.1 (C-9 and C-10), 23.9 (C-5), 27.8 (C-6), 47.5 (C-7), 50.1 (C-

8), 56.0 (C-4), 64.2 (C-1), 73.4 (C-3), 85.5 and 89.0 (C≡C), 121.2, 128.4, 129.3 and 131.9 (Ph), 194.0 (C-2).

b) Reaction with 1-heptyne:

Yield is 35 %, **12b**:**13b** = 90:10. Anal. Calcd for $C_{17}H_{25}NO_3S$: C, 63.13; H, 7.79; N, 4.33, S, 9.91. Found: C, 62.99; H, 7.99; N, 4.14; S, 9.76. CI-MS, *m/z*: (M is 323.458) 324 $[M + H]^+$, 260 $[M - SO_2]^+$.

(3a*S*,7a*S*)-7a-Heptynyl-8,8-dimethyl-7-oxo-1,4,5,6,7,7a-hexahydro-3*H*-3a,6-methano-2,1-benzisothiazole 2,2-dioxide (12b)

Colourless solid. TLC on SiO_2, R_f = 0.42 (eluent CH_2Cl_2/Et_2O 9:1). IR spectrum (selected bands), cm^{-1}: 3217 s ν(NH), 2227 w ν (C≡C), 1764 s ν (C=O), 1314 and 1142 s ν (SO_2). ^1H NMR spectrum (500 MHz) in $CDCl_3$, δ (ppm): 0.81 (t, 7.1 Hz, 3H, heptynyl-7), 1.02 (s, 3H, H-10), 1.21 (s, 3H, H-9), 1.29 (m, 4H, heptynyl-6 and -5), 1.45 (quint., J = 7.2 Hz, 2H, heptynyl-4), 1.75 (m, 1H, H-5 endo), 1.99 (m, 2H, H-6 and H-5 exo), 2.18 (t, J = 7.2 Hz, 2H, heptynyl-3), 2.39 (m, 1H, H-6), 2.38 (m, 1H, H-4), 3.32 (“s”, 2H, H-8), 5.15 (s, br., 1H, NH). ^{13}C NMR spectrum (125 MHz) in $CDCl_3$, δ (ppm): 14.2 (CH_3 heptynyl-7), 19.1 (CH_2 heptynyl-3), 20.0 (C-10), 21.9 (C-5), 22.3 (CH_2 heptynyl-6), 22.8 (C-9), 28.1 (CH_2 heptynyl-4), 29.2 (C-6), 31.2 (CH_2 heptynyl-5), 45.2 (C-7), 49.9 (C-8), 57.0 (C-1), 58.7 (C-4), 65.6 (C-2), 75.7 and 91.7 (C≡C), 206.3 (C-3).

(3a*S*,7*R*)-7-Heptynyl-7-hydroxy-8,8-dimethyl-4,5,6,7-tetrahydro-3*H*-3a,6-methano-2,1-benzisothiazole 2,2-dioxide (13b)

Colourless solid. TLC on SiO_2, R_f = 0.40 (eluent CH_2Cl_2/Et_2O 9:1). IR spectrum (selected bands), cm^{-1}: 3452 s ν(OH), 2227 w ν (C≡C), 1653 s ν (C=N), 1328 and 1155 s ν (SO_2). ^1H NMR spectrum (400 MHz) in $CDCl_3$, δ (ppm): 0.82 (t, J = 7.2 Hz, 3H, heptynyl-7), 1.00 (s, 3H, H-10), 1.02 (s, 3H, H-9), 1.30 (m, 4H, heptynyl-6 and -5), 1.46 (m, 2H, heptynyl-4), 1.68 (m, 1H, H-5), 1.91 (m, 1H, H-6), 1.93 (m, 1H, H-5), 2.20 (t, J = 7.4 Hz, 2H, heptynyl-3), 2.04 (m, 1H, H-6), 2.15 (m, 1H, H-4), 3.08 (d, J = 13.4 Hz, 1H, H-8),

3.18 (d, J = 13.4 Hz, 1H, H-8), 3.20 (s, 1H, OH). ^{13}C NMR spectrum (100 MHz) in $CDCl_3$, δ (ppm): 14.0 (CH_3 heptynyl-7), 19.0 (CH_2 heptynyl-3), 21.0 and 21.1 (C-9 and C-10), 22.2 (CH_2 heptynyl-6), 23.8 (C-5), 27.9 (C-6), 27.9 (CH_2 heptynyl-4), 31.2 (CH_2 heptynyl-5), 47.4 (C-7), 50.0 (C-8), 55.9 (C-4), 64.2 (C-1), 73.0 (C-3), 76.3 and 90.2 (C≡C), 194.4 (C-2).

c) Reaction with 1-adamantylacetylene:

Yield is 88 %, **12c**:**13c** = 70:30. Anal. Calcd for $C_{22}H_{29}NO_3S$: C, 68.18; H, 7.54; N, 3.61, S, 8.27. Found: C, 68.00; H, 7.89; N, 3.53; S, 8.11. EI-MS, *m/z*: (M is 387.545) 388 $[M + H]^+$, 324 $[M - SO_2]^+$, 135 $[adamantyl]^+$.

(3a*S*,7a*S*)-7a-(1-Adamantylethynyl)-8,8-dimethyl-7-oxo-1,4,5,6,7,7a-hexahydro-3*H*-3a,6-methano-2,1-benzisothiazole 2,2-dioxide (12c)

Colourless solid. TLC on SiO_2, R_f = 0.48 (eluent CH_2Cl_2/Et_2O 9:1). IR spectrum (selected bands), cm^{-1}: 3221 s ν(NH), 2230 w ν(C≡C), 1764 s ν(C=O), 1316 and 1145 s ν (SO_2). ^{1}H NMR spectrum (400 MHz) in $CDCl_3$, δ (ppm): 1.09 (s, 3H, H-10), 1.27 (s, 3H, H-9), 1.64 (m, br., 6H), 1.86 (m, br., 6H) and 2.00 (m, br., 3H)(adamantyl), 1.78 (m, 1H, H-5endo), 2.01 (m, 1H, H-6), 2.06 (m, 1H, H-5exo), 2.42 (m, 1H, H-6), 2.40 (d, J = 5.0 Hz, 1H, H-4), 3.37 (“s”, 2H, H-8), 4.73 (s, br., 1H, NH). ^{13}C NMR spectrum (100 MHz) in $CDCl_3$, δ (ppm): 19.8 (C-10), 21.8 (C-9), 22.7 (C-5), 27.7 (ada CH), 29.2 (C-6), 29.9 (ada C_q), 36.2 and 42.3 (ada CH_2), 45.0 (C-7), 49.6 (C-8), 56.7 (C-1), 58.5 (C-4), 64.9 (C-2), 74.4 and 99.0 (C≡C), 206.2 (C-3).

(3a*S*,7*R*)-7-(1-Adamantylethynyl)-7-hydroxy-8,8-dimethyl-4,5,6,7-tetrahydro-3*H*-3a,6-methano-2,1-benzisothiazole 2,2-dioxide (13c)

Colourless solid. TLC on SiO_2, R_f = 0.45 (eluent CH_2Cl_2/Et_2O 9:1). IR spectrum (selected bands), cm^{-1}: 3480 s ν(OH), 2230 w ν(C≡C), 1652 s ν (C=N), 1326 and 1158 s ν (SO_2). ^{1}H NMR spectrum (400 MHz) in $CDCl_3$, δ (ppm): 1.00 (s, 3H) and 1.09 (s, 3H)(H-9 and H-10), 1.66 (m, 1H, H-6), 1.90 (m, 1H, H-5exo), 1.93 (m, 1H, H-6), 2.03 (m, 1H, H-5endo),

1.62 (m, br., 6H), 1.88 (m, br., 6H) and 2.01 (m, br., 3H)(adamantyl), 2.17 (d, J = 3.8 Hz, 1H, H-4), 3.13 (d, J = 13.4 Hz, 1H, H-8), 3.21 (d, J = 13.4 Hz, 1H, H-8), 3.18 (s, br., OH). ^{13}C NMR spectrum (100 MHz) in $CDCl_3$, δ (ppm): 21.0 and 21.1 (C-9 and C-10), 24.0 (C-5), 27.6 (ada CH), 28.0 (C-6), 29.8 (ada C_q), 36.2 and 42.0 (ada CH_2), 47.4 (C-7), 50.1 (C-8), 55.9 (C-4), 64.2 (C-1), 73.0 (C-3), 76.1 and 98.3 (C≡C), 194.9 (C-2).

d) Reaction with 1-methoxy-1-ethynyl-cyclohexane:

Yield is 67 %, **12d**:**13d** = 50:50. Anal. Calcd for $C_{19}H_{27}NO_4S$: C, 62.44; H, 7.45; N, 3.83, S, 8.77. Found: C, 61.98; H, 7.52; N, 3.77; S, 8.89. EI-MS, *m/z*: (M is 365.495) 366 $[M + H]^+$, 348 $[M - H_2O]^+$, 334 $[M - OMe]^+$, 270 $[M - OMe - SO_2]^+$.

(3a*S*,7a*S*)-7a-(1-Methoxycyclohexyl)ethynyl-8,8-dimethyl-7-oxo-1,4,5,6,7,7a-hexahydro-3*H*-3a,6-methano-2,1-benzisothiazole 2,2-dioxide (12d)

Colourless solid. TLC on SiO_2, R_f = 0.50 (eluent CH_2Cl_2/Et_2O 9:1). IR spectrum (selected bands), cm^{-1}: 3217 s ν(NH), 2228 w ν (C≡C), 1766 s ν(C=O), 1317 and 1143 s ν (SO_2). ^1H NMR spectrum (400 MHz) in $CDCl_3$, δ (ppm): 0.98 (s, 3H, H-10), 1.16 (s, 3H, H-9), 1.65 (m, 1H, H-5 endo), 1.93 (m, 1H, H-6), 1.97 (m, 1H, H-5 exo), 2.30 (m, 1H, H-6), 2.33 (d, J = 4.8 Hz, 1H, H-4), 3.22 (s, 3H, OMe), 3.26 ("s", 2H, H-8), 1.18 (m, 2H), 1.38 (m, 2H), 1.47 (m, 2H), 1.55 (m, 2H), 1.80 (m, 2H)(cyclohexyl), 5.01 (s, br., 1H, NH). ^{13}C NMR spectrum (100 MHz) in $CDCl_3$, δ (ppm): 19.7 (C-10), 22.0 (C-5), 22.6 (C-9), 29.1 (C-6), 45.1 (C-7), 49.6 (C-8), 51.0 (OMe), 56.8 (C-1), 58.3 (C-4), 64.7 (C-2), 205.4 (C-3), 22.7, 25.3 and 36.4 (cyclohexyl CH_2), 74.2 (cyclohexyl C_q), 81.4 and 91.5 (C≡C).

(3a*S*,7*R*)-7-(1-Methoxycyclohexyl)ethynyl-8,8-dimethyl-7-hydroxy-4,5,6,7-tetrahydro-3*H*-3a,6-methano-2,1-benzisothiazole 2,2-dioxide (13d)

Colourless solid. TLC on SiO_2, R_f = 0.48 (eluent CH_2Cl_2/Et_2O 9:1). IR spectrum (selected bands), cm^{-1}: 3444 s ν(OH), 2228 w ν (C≡C), 1655 s ν (C=N), 1322 and 1160 s ν (SO_2). ^1H NMR spectrum (400 MHz) in $CDCl_3$, δ (ppm): 0.99 (s, 3H) and 1.00 (s, 3H)(H-9 and H-10), 1.68 (m, 1H, H-6), 1.90 (m, 1H, H-5 exo), 1.93 (m, 1H, H-6), 2.03 (m, 1H, H-5

endo), 2.17 (d, J = 3.8 Hz, 1H, H-4), 3.02 (d, J = 13.5 Hz, 1H, H-8), 3.12 (d, J = 13.4 Hz, 1H, H-8), 3.22 (s, 3H, OMe), 1.17 (m, 2H), 1.39 (m, 2H), 1.48 (m, 2H), 1.54 (m, 2H), 1.82 (m, 2H)(cyclohexyl), 3.23 (s, br., 1H, OH). ^{13}C NMR spectrum (100 MHz) in $CDCl_3$, δ (ppm): 21.0 and 21.1 (C-9 and C-10), 23.9 (C-5), 28.0 (C-6), 47.5 (C-7), 49.9 (C-8), 51.0 (OMe), 55.8 (C-4), 64.1 (C-1), 72.9 (C-3), 194.5 (C-2), 22.8, 25.2 and 36.5 (cyclohexyl CH_2) and 74.0 (cyclohexyl C_q), 83.0 and 90.7 (C≡C).

Reduction pathway

(3a*S*,7a*S*)-8,8-Dimethyl-7-oxo-1,4,5,6,7,7a-hexahydro-3*H*-3a,6-methano-2,1-benzisothiazole 2,2-dioxide (3-oxo-camphorsultam) (14)

To a well-stirred solution of 3-oxo-camphorsulfonimide (**3**, 2.29 g, 10 mmol) in acetic acid (20 mL) and hot water (150 mL), zinc powder (1.4 g, 20 mmol) was added in small portions over a period of 1 h. The reaction mixture was kept at 80 °C for another hour. Then, the excess of zinc powder was filtered off and the solvent was evaporated. The resulting white solid was extracted with chloroform to separate the organic material from the insoluble zinc salts. The filtrate was evaporated and the residue recrystallised from chloroform/diethyl ether.

Yield is 65 %. Anal. Calcd for $C_{10}H_{15}NO_3S$: C, 52.38; H, 6.59; N, 6.11, S, 13.98. Found: C, 52.24; H, 6.51; N, 6.19; S, 14.11. EI-MS, *m/z*: (M is 229.300) 229 $[M]^+$, 201 $[M - CO]^+$, 132 $[M - 97]^+$. M.p. 170 ^{o}C. IR spectrum (selected bands), cm^{-1}: 3180 w ν(N–H), 1760 s ν(C=O), 1310 and 1130 s ν(SO_2). ^{1}H NMR spectrum (400 MHz) in $CDCl_3$, δ (ppm): 1.03 (s, 3H) and 1.15 (s, 3H)(9-H and 10-H), 1.73 (m, 1H), 1.84 (m, 1H) and 2.05-2.20 (m, 2H)(5-H and 6-H), 2.36 (d, J = 3.8 Hz, 1H, 4-H), 3.32 (d, J = 13.8 Hz, 1H) and 3.34 (d, J = 13.8 Hz, 1H)(8-H), 3.58 (d, J = 4.3 Hz, 1H, 2-H), 5.57 (d, J = 4.3 Hz, 1H, NH). ^{13}C NMR spectrum (100 MHz) in $CDCl_3$, δ (ppm): 18.8 and 20.9 (C-9 and C-10), 20.4 and 30.85 (C-5 and C-6), 45.7 (C-7), 49.4 (C-8), 52.4 (C-1), 57.6 (C-4), 65.0 (C-2), 209.7 (C-3).

(3a*S*,7*R*,7a*S*)-7-Hydroxy-8,8-dimethyl-7-phenylethynyl-1,4,5,6,7,7a-hexahydro-3*H*-3a,6-methano-2,1-benzisothiazole 2,2-dioxide (3-exo-hydroxy-3-endo-phenylethinyl-camphorsultam) (15)

Similar as described in [4], a solution of phenylacetylene (1.1 g, 10.8 mmol) in dry diethyl ether (10 mL) was cooled in an ice bath. Butyl lithium (1.6 M in hexanes, 6.25 mL, 10 mmol) was added and the reaction mixture was left at room temperature for 30 min, before it was added dropwise to a suspension of 3-oxo-camphersultam (**14**, 1.15 g, 5 mmol) in dry diethyl ether (10 mL). The reaction mixture was stirred overnight, water (10 mL) was added and the organic phase was separated. The aqueous phase was extracted twice with dichloromethane, and the combined organic phases were dried with Na_2SO_4. After chromatography on SiO_2 (eluent CH_2Cl_2/Et_2O 9:1) the compound was obtained as a white solid.

Yield is 72 %. Anal. Calcd for $C_{18}H_{21}NO_3S$: C, 65.23; H, 6.39; N, 4.23, S, 9.67. Found: C 65.17; H, 6.44; N, 4.11; S, 9.73. CI-MS, *m/z*: (M is 331.437) 332 $[M + H]^+$. M.p. 74-76 ^oC. TLC on SiO_2, R_f = 0.48 (eluent CH_2Cl_2/Et_2O 9:1). IR spectrum (selected bands), cm^{-1}: 3292 s ν(N–H), 2218 w ν(C≡C), 1308 and 1133 s $\nu(SO_2)$. ^1H NMR spectrum (400 MHz) in $CDCl_3$, δ (ppm): 0.96 (s, 3H) and 1.35 (s, 3H)(9-H, 10-H), 1.45 (m, 1H), 1.86-1.94 (m, 2H) and 2.02 (m, 1H)(5-H and 6-H), 2.16 (d, J = 4.8 Hz, 1H, 4-H), 3.07 (s, br., 1H, OH), 3.19 (s, 2H, 8-H), 3.67 (d, J = 9.9 Hz, 2-H), 4.86 (d, J = 9.9 Hz, NH), 7.33 (m, 3H) and 7.43 (m, 2H)(Ph). ^{13}C NMR spectrum (100 MHz) in $CDCl_3$, δ (ppm): 22.1 and 22.8 (C-9 and C-10), 23.8 and 30.3 (C-5 and C-6), 48.9 (C-7), 51.2 (C-8), 55.8 (C-4), 57.5 (C-1), 73.6 (C-2), 76.0 (C-3), 85.3 and 90.9 (C≡C), 121.7, 128.4, 128.8 and 131.7 (Ph).

Acetal pathway:

(3a*S*,7a*S*)-7,7-Dimethoxy-8,8-dimethyl-7a-phenylethynyl-1,4,5,6,7,7a-hexahydro-3*H*-3a,6-methano-2,1-benzisothiazole 2,2-dioxide (2-endo-phenylethinyl-3,3-dimethoxy-camphorsultam) (17a)

Similar as described in [4], a solution of the phenylacetylene (520 mg, 5.1 mmol) in dry diethyl ether (10 mL) was cooled in an ice bath. Butyl lithium (1.6 M in hexanes, 6.25 mL, 5 mmol) was added and the reaction mixture was left at room temperature for 30 min, before it was added dropwise to a suspension of 3,3-dimethoxy-camphorsulfonylimine (**16**) [3] (1.37 g, 5 mmol) in dry diethyl ether (10 mL). The reaction mixture was stirred overnight, water (10 mL) was added and the organic phase was separated. The aqueous phase was extracted twice with dichloromethane, and the combined organic phases were dried with Na_2SO_4. After chromatography on SiO_2 (eluent CH_2Cl_2/Et_2O 9:1) the compound was obtained as a colourless solid.

Yield is 82 %. Anal. Calcd for $C_{20}H_{25}NO_4S$: C, 63.98; H, 6.71; N, 3.73, S, 8.54. Found: C 64.08; H, 6.79; N, 3.67; S, 8.66. EI-MS, *m/z*: (M is 375.491) 375 $[M]^+$, 360 $[M - NH]^+$, 345 $[M - OMe]^+$, 311 $[M - SO_2]^+$, 183 $[M - 192]^+$, 101 $[PhCCH]^+$, 77 $[Ph]^+$. M.p. 217 ^oC. TLC on SiO_2, R_f = 0.31 (eluent CH_2Cl_2). IR spectrum (selected bands), cm^{-1}: 3348 ν(NH), 2231 w ν(C≡C), 1307 and 1125 s ν (SO_2). ^1H NMR spectrum (500 MHz) in $CDCl_3$, δ (ppm): 0.99 (s, 3H) and 1.43 (s, 3H)(9-H, 10-H), 1.72-1.82 (m, 2H), 2.06 (m, 1H) and 2.36 (m, 1H)(5-H and 6-H), 2.23 (d, J = 4.8Hz, 1H, 4-H), 3.22 (d, J = 14.0 Hz, 1H) and 3.27 (d, J = 14.0 Hz, 1H)(8-H), 3.32 (s, 3H) and 3.41 (s, 3H)(2 OMe), 5.20 (s, 1H, NH), 7.27-7.33 (m, 3H) and 7.43-7.49 (m, 2H)(Ph). ^{13}C NMR spectrum (125 MHz) in $CDCl_3$, δ (ppm): 21.7 and 23.1 (C-9 and C-10), 20.5 and 28.6 (C-5 and C-6), 46.6 (C-7), 49.3 (C-4), 50.0 (C-8), 50.9 and 51.2 (2 OMe), 63.4 (C-1), 70.8 (C-2), 86.7 and 88.2 (C≡C), 108.7 (C-3), 122.4, 128.1, 128.3 and 131.5 (Ph).

(3a*S*,7a*S*)-7,7-Diethoxy-8,8-dimethyl-7a-phenylethynyl-1,4,5,6,7,7a-hexahydro-3*H*-3a,6-methano-2,1-benzisothiazole 2,2-dioxide (2-endo-phenylethinyl-3,3-diethoxy-camphorsultam) (17a')

The compound was prepared in analogy to **17a** from 3,3-diethoxy-camphorsulfonylimine (**16'**) [3] as a starting material. Yield is 77%. Anal. Calcd for $C_{22}H_{29}NO_4S$: C, 65.48; H,

7.24; N, 3.47, S, 7.93. Found: C 65.08; H, 7.11; N, 3.55; S, 8.11. EI-MS, *m/z*: (M is 403.545) 404 $[M]^+$, 359 $[M - OEt]^+$, 340 $[M - SO_2]^+$, 101 $[PhCCH]^+$, 77 $[Ph]^+$. TLC on SiO_2, $R_f = 0.33$ (eluent CH_2Cl_2). IR spectrum (selected bands), cm^{-1}: 3411 ν(NH), 2233 w ν(C≡C), 1305 and 1123 s ν (SO_2). ^1H NMR spectrum (500 MHz) in $CDCl_3$, δ (ppm): 1.23 (t, 7.2 Hz, 6H, 2 OEt), 1.00 (s, 3H) and 1.43 (s, 3H)(9-H, 10-H), 1.73-1.84 (m, 2H), 2.06 (m, 1H) and 2.37 (m, 1H)(5-H and 6-H), 2.25 (d, J = 4.8Hz, 1H, 4-H), 3.22 (d, J = 14.0 Hz, 1H) and 3.26 (d, J = 14.0 Hz, 1H)(8-H), 3.44 (m, 1H), 3.57 (m, 2H) and 3.95 (m, 1H)(2 OEt), 5.19 (s, 1H, NH), 7.29-7.33 (m, 3H) and 7.45-7.48 (m, 2H)(Ph). ^{13}C NMR spectrum (125 MHz) in $CDCl_3$, δ (ppm): 14.2 and 14.6 (2 OEt), 21.7 and 23.2 (C-9 and C-10), 20.5 and 28.6 (C-5 and C-6), 46.5 (C-7), 49.5 (C-4), 50.0 (C-8), 58.5 and 59.1 (2 OEt), 63.3 (C-1), 70.7 (C-2), 86.8 and 88.2 (C≡C), 108.7 (C-3), 122.4, 128.0, 128.1 and 131.4 (Ph).

(3a*S*,7a*S*)-7,7-Dimethoxy-8,8-dimethyl-7a-(1-heptynyl)-1,4,5,6,7,7a-hexahydro-3*H*-3a,6-methano-2,1-benzisothiazole 2,2-dioxide (2-endo-heptynyl-3,3-dimethoxy-camphorsultam) (17b)

The compound was prepared in analogy to **17a** by reaction of 3,3-dimethoxy-camphorsulfonylimine (**16**) with 1-heptynyl lithium. Yield 41%. Accurate EI-MS, m/z: Calcd for $C_{19}H_{32}NO_4S$ $[M + H]^+$: 370.2040. Found: 370.2047 (Delta [mmu] -0.7). EI-MS, *m/z*: 370 $[M + H]^+$, 338 $[M - MeOH]^+$, 306 $[M - SO_2]^+$, 279 $[M - MeOH - SO_2]^+$. IR spectrum (selected bands), cm^{-1}: 3360 ν(NH), 2252 w ν(C≡C), 1306 and 1123 s ν(SO_2). ^1H NMR spectrum (500 MHz) in $CDCl_3$, δ (ppm): 0.90 (t, 3H, J = 7.2 Hz, 3H, heptynyl-7), 0.98 (s, 3H, H-10), 1.33 (m, 2H, heptynyl-6), 1.41 (m, 2H, heptynyl-5), 1.40 (s, 3H, H-9), 1.53 (quint., J = 7.3 Hz, 2H, heptynyl-4), 1.73 (m, 2H, H-5 exo and H-6 exo), 1.98 (m, 1H, H-5 endo), 2.27 (d, J = 5.0 Hz, 1H, H-4), 2.28 (t, J = 7.3 Hz, 2H, heptynyl-13), 2.31 (1H, m, H-6 endo), 3.18 (d, J = 14.2 Hz, 1H, H-8), 3.19 (d, J = 14.2 Hz, 1H, H-8), 3.28 (s, 3H, OMe exo), 3.35 (s, 3H, OMe endo), 5.01 (s, br., 1H. NH). ^{13}C NMR spectrum (125 MHz) in $CDCl_3$, δ (ppm): 14.2 (CH_3, heptynyl-7), 19.2 (CH_2, heptynyl-3), 20.8 (CH_2, C-5), 22.2

(CH_3, C-9), 22.3 (CH_2, heptynyl-6), 23.6 (CH_3, C-10), 28.2 (CH_2, heptynyl-4), 29.0 (CH_2, C-6), 31.2 (CH_2, heptynyl-5), 46.7 (C_q, C-7), 49.7 (CH, C-4), 50.4 (CH_2, C-8), 51.2 (CH_3, OMe exo), 51.5 (CH_3, OMe endo), 63.7 (C_q, C-1), 70.9 (C_q, C-2), 77.7 (C_q) and 89.7 (C_q)(C≡C), 108.8 (C_q, C-3).

(3a*S*,7a*S*)-8,8-Dimethyl-7-oxo-7a-phenylethynyl-1,4,5,6,7,7a-hexahydro-3*H*-3a,6-methano-2,1-benzisothiazole 2,2-dioxide (12a)

To a solution of **17a** or **17a'** (4 mmol) in acetone (5 mL), conc. HCl (0.05 mL) was added and the reaction mixture was stirred at room temperature overnight. After evaporation of the solvent and chromatography (SiO_2, CH_2Cl_2/Et_2O 9:1) the compound was obtained as a colourless solid. Yield is 91% (from **17a**); 82% (from **17a'**). For analytical data of the product **12a**, see above.

(3a*S*,7a*S*)-8,8-Dimethyl-7-oxo-7a-(1-heptynyl)-1,4,5,6,7,7a-hexahydro-3*H*-3a,6-methano-2,1-benzisothiazole 2,2-dioxide (12b)

This compound was prepared in analogy to **12a** from **17b** as starting material and obtained as colourless oil with 81% yield. For analytical data of product **12b**, see above.

(3a*S*,7*R*,7a*S*)-7-Hydroxy-8,8-dimethyl-7a-phenylethynyl-1,4,5,6,7,7a-hexahydro-3*H*-3a,6-methano-2,1-benzisothiazole 2,2-dioxide (2-endo-phenylethinyl-3-exo-hydroxy-camphorsultam) (18)

A solution of **12a** (200 mg, 0.61 mmol) in ethanol (5 mL) was cooled to 0 °C. Then, solid $NaBH_4$ (28 mg, 0.74 mmol) was added, the ice bath was removed and the reaction mixture stirred at room temperature for 15 min. Water (5 mL) was added and the reaction mixture was heated to reflux for 15 min. The mixture was extracted with diethylether (5 mL) and then with CH_2Cl_2 (2 × 3 mL) and the organic phase was dried over $MgSO_4$. After evaporation of the solvent and chromatography (SiO_2, CH_2Cl_2/Et_2O 9:1) the product was obtained as a colourless solid.

Yield is 86%. Accurate EI-MS, m/z: Calcd for $C_{18}H_{20}NO_3S$ $[M - H]^+$: 330.1164. Found: 330.1174 (Δ [mmu] –1.0). EI-MS, *m/z*: (M is 331.437) 331 $[M]^+$, 330 $[M - H]^+$, 267 $[M - SO_2]^+$, 102 $[PhCCH]^+$. M.p. 202-203 °C. TLC on SiO_2, R_f = 0.42 (CH_2Cl_2/Et_2O 9:1). IR spectrum (selected bands), cm^{-1}: 3524 and 3452 s ν(O–H), 3350 and 3297 ν(N–H), 2217 w ν(C≡C), 1305 and 1128 s ν(SO_2). ^1H NMR spectrum (500 MHz) in $CDCl_3$, δ (ppm): 0.99 (s, 3H, 9/10-H), 1.44 (s, 3H, 9/10-H), 1.34 (m, 1H, 5-H endo), 1.81 (m, 1H, 6-H endo), 1.92 (m, 1H, 5-H exo), 2.23 (m, 1H, 6-H exo), 2.02 (d, J = 5.2 Hz, 1H, 4-H), 2.69 (d, J = 6.0 Hz, 1H, OH), 3.32 (d, J = 13.6Hz, 1H, H-8), 3.40 (d, J = 13.6 Hz, 1H, H-8), 4.31 (d, J = 6.4 Hz, 1H, H-3 endo), 4.97 (s, 1H, NH), 7.31 (m, 3H) and 7.44 (m, 2H)(Ph). ^{13}C NMR spectrum (125 MHz) in $CDCl_3$, δ (ppm): 22.8 (CH_3) and 23.4 (CH_3)(C-9,C-10), 24.4 (CH_2, C-5), 28.5 (CH_2, C-6), 49.4 (C_q, C-7), 51.3 (CH_2, C-8), 51.4 (CH, C-4), 61.6 (C_q, C-1), 68.7 (C_q, C-2), 85.6 (CH, C-3), 86.9 and 89.6 (C≡C), 122.3 (C_q), 128.5, 128.9 and 132.1(CH)(Ph).

Imine pathway:

(3a*S*)-8,8-Dimethyl-7-(2-phenylethyl)imino-4,5,6,7-tetrahydro-3*H*-3a,6-methano-2,1-benzisothiazole 2,2-dioxide (3-((2-phenylethyl)imino)-camphorsulfonylimine) (19) [5]

A solution of 3-oxo-camphorsulfonylimine (**3**, 10.6 g, 50 mmol) and 2-phenylethylamine (21.2 g, 175 mmol) in toluene (200 mL) was cooled to 0 °C. A solution of $TiCl_4$ (6.5 g, 25 mmol) in toluene (50 mL) was added dropwise. The reaction mixture was refluxed for 16 h and then cooled to room temperature. Chloroform (250 mL) was added and the mixture stirred for 2 h. After filtration, activated charcoal (2 g) was added to the filtrate. After stirring for 10 min, the charcoal was filtered off over a bed of Celite, the solvent was evaporated and the residual solid recrystallised from chloroform/diethyl ether.

Yield is 61.7%. Accurate EI-MS, m/z: Calcd for $C_{18}H_{22}N_2O_2S$ $[M]^+$: 330.1402. Found: 330.1418 (Δ [mmu] –1.6). EI-MS, *m/z*: (M is 330.452) 330 $[M]^+$, 266 $[M - SO_2]^+$, 105

$[PhCH_2CH_2]^+$. M.p. 148-150 °C. TLC on SiO_2, R_f = 0.66 (eluent ethyl acetate/hexane 2:1). IR spectrum (selected bands), cm^{-1}: 1675 and 1647 s ν(C=N), 1336 and 1160 s ν(SO_2). ^1H NMR spectrum (500 MHz) in $CDCl_3$, δ (ppm): 0.65 (s, 3H) and 1.01 (s, 3H)(9-H, 10-H), 1.09 (m, 1H, H-5 endo), 1.77 (m, 1H, H-6 endo), 1.95 (m, 1H, H-5 exo), 2.06 (m, 1H, H-6 exo), 2.85 (d, J = 4.8 Hz, H-4), 3.07 (d, J = 12.0 Hz, 1H) and 3.28 (d, J = 12.0 Hz, 1H)(H-8), 3.09 (m, 2H, CH_2Ph), 3.91 (m, 2H, CH_2N=), 7.21 (m, 3H) and 7.26 (m, 2H)(Ph). ^{13}C NMR spectrum (125 MHz) in $CDCl_3$, δ (ppm): 18.3 and 19.4 (C-9 and C-10), 23.1 (C-5), 28.3 (C-6), 36.3 (CH_2Ph), 46.0 (C-7), 49.6 (C-4), 49.7 (C-8), 57.2 (CH_2N=), 62.6 (C-1), 126.4, 128.4, 128.9 and 139.4 (Ph), 167.0 (C-3), 185.0 (C-2).

(3a*S*,7a*S*)-8,8-Dimethyl-7-(2-phenylethyl)imino-7a-phenylethynyl-1,4,5,6,7,7a-hexahydro-3*H*-3a,6-methano-2,1-benzisothiazole 2,2-dioxide (20)

Similar as described in [4], a solution of phenylacetylene (520 mg, 5.1 mmol) in dry diethyl ether (10 mL) was cooled in an ice bath. Butyl lithium (1.6 M in hexanes, 6.25 mL, 5 mmol) was added dropwise and the reaction mixture was left at room temperature for 30 min, before it was added dropwise to a suspension of 3-((2-phenylethyl)imino)-camphorsulfonylimine (**19**, 1.65 g, 5 mmol) in dry diethyl ether (10 mL). The reaction mixture was stirred overnight, water (10 mL) was added and the organic phase was separated. The aqueous phase was extracted twice with dichloromethane, and the combined organic phases were dried with Na_2SO_4. After chromatography on SiO_2 (eluent CH_2Cl_2/Et_2O 9:1) the compound was obtained as a pale yellow solid.

Yield is 78%. Accurate EI-MS, m/z: Calcd for $C_{26}H_{27}N_2O_2S$ $[M - H]^+$: 431.1793. Found: 431.1793 (Δ [mmu] 0.0). EI-MS, *m/z*: (M is 432.589) 432 $[M]^+$, 431 $[M – H]^+$, 368 $[M – SO_2]^+$, 328 $[M – PhCH=CH_2]^+$, 264 $[M – SO_2 – PhCH=CH_2]^+$, 105 $[PhCH_2CH_2]^+$, 102 $[PhCCH]^+$. M.p. 87-88 °C. TLC on SiO_2, R_f = 0.56 (eluent ethyl acetate/hexane 2:1). IR spectrum (selected bands), cm^{-1}: 3195 s ν(N–H), 2221 w ν(C≡C), 1695 s ν(C=N), 1309 and 1144 s ν(SO_2). ^1H NMR spectrum (500 MHz) in $CDCl_3$, δ (ppm): 1.00 (s, 3H, H-10),

1.14 (s, 3H, H-9), 1.02 (m, 1H, H-5 endo), 1.73 (m, 1H, H-5 exo), 1.91 (td, J = 12.0 Hz, J = 6.0 Hz, 1H, H-6 exo), 2.36 (m, 1H, H-6 endo), 2.91 (m, 1H) and 3.00 (m, 1H)(CH_2Ph), 2.71 (d, J = 4.6 Hz, 1H, H-4), 3.30 (d, J = 12.2 Hz, 1H, H-8 syn), 3.37 (d, J = 12.2 Hz, 1H, H-8 anti), 3.67 (m, 1H) and 3.76 (m, 1H)(CH_2N=), 7.21 (m, 3H) and 7.26 (m, 2H)(CH_2*Ph*), 7.15 (m, 1H), 7.30 (m, 2H) and 7.47 (d, 8.2 Hz, 2H)(PhC≡C). ^{13}C NMR spectrum (125 MHz) in $CDCl_3$, δ (ppm): 20.2 (C-10), 22.6 (C-5), 22.8 (C-9), 29.5 (C-6), 36.8 (*C*H_2Ph), 46.7 (C-7), 48.9 (C-4), 50.0 (C-8), 55.4 (CH_2N=), 57.6 (C-1), 66.1 (C-2), 87.3 and 88.0 (C≡C), 122.5, 126.5, 128.4 and 128.6 (CH_2*Ph*), 128.9, 129.4, 132.2 and 140.0 (*Ph*C≡C), 175.8 (C-3).

(3a*S*,7a*S*)-8,8-Dimethyl-7-oxo-7a-phenylethynyl-1,4,5,6,7,7a-hexahydro-3*H*-3a,6-methano-2,1-benzisothiazole 2,2-dioxide (12a)

To a suspension of **20** (107 mg, 0.25 mmol) in water (5 mL), conc. HCl (0.5 mL) was added and the reaction mixture was refluxed overnight. The reaction mixture was left to cool and the crude product was collected by filtration. The product was purified by chromatography (SiO_2, CH_2Cl_2/Et_2O 9:1) and obtained as a colourless solid. Yield is 38%. For analytical data of product **12a**, see above.

Bisalkyne derivatives:

(3a*S*,7*R*,7a*S*)-8,8-Dimethyl-7-heptynyl-7-hydroxy-7a-phenylethynyl-1,4,5,6,7,7a-hexahydro-3*H*-3a,6-methano-2,1-benzisothiazole 2,2-dioxide (2-endo-phenylethinyl-3-endo-heptynyl-3-exo-hydroxy-camphorsultam) (21a)

Similar as described in [4], a solution of 1-heptyne (395 mg, 4.1 mmol) in dry diethyl ether (5 mL) was cooled in an ice bath. Butyl lithium (1.6 M in hexanes, 2.5 mL, 4 mmol) was added and the reaction mixture was left at room temperature for 30 min, before it was added dropwise to a suspension of **12a** (660 mg, 2 mmol) in dry diethyl ether (5 mL). The reaction mixture was refluxed overnight, water (10 mL) was added and the organic phase was separated. The aqueous phase was extracted twice with dichloromethane and the

combined organic phases were dried with Na_2SO_4. After chromatography on SiO_2 (eluent CH_2Cl_2/Et_2O 9:1) the compound was obtained as a white solid.

Yield 76%. Elemental analysis calculated for $C_{25}H_{31}NO_3S$: C 70.55; H 7.34; N 3.29; found: C 70.44; H 7.69; N 3.10. EI-MS: (M = 425.595) 426 $[M + H]^+$, 408 $[M - H_2O]^+$, 330 $[M - \text{heptyne}]^+$, 324 $[M - PhC{\equiv}CH]^+$. IR spectrum (selected bands), cm^{-1}: 3411 s ν(OH), 3323 ν(NH), 2233 w ν(C≡C), 1335 and 1149 s $\nu(SO_2)$. ^{1}H NMR spectrum (500 MHz) in $CDCl_3$, δ (ppm): 0.73 (t, 7.5 Hz, 3H, heptynyl-7), 0.95 (s, 3H, H-10), 1.13 (m, 2H, heptynyl-6), 1.22 (m, 2H, heptynyl-5), 1.38 (m, 2H, heptynyl-4), 1.41 (s, 3H, H-9), 1.71 (m, 1H, H-6 exo), 1.79 (m, 1H, H-5 exo), 1.97 (m, 1H, H-5 endo), 2.03 (d, J = 5.1 Hz, 1H, H-4), 2.16 (t, J = 7.2 Hz, 2H, heptynyl-3), 2.19 (m, 1H, H-6 endo), 2.87 (s, br., 1H, OH), 3.25 (d, J = 13.5 Hz, 1H, H-8 syn), 3.30 (d, J = 13.5 Hz, 1H, H-8 anti), 5.08 (s, 1H, NH), 7.22 (m, 3H, *m*- and *p*-Ph), 7.40 (m, 2H, *o*-Ph). ^{13}C NMR spectrum (125 MHz) in $CDCl_3$, δ (ppm): 14.1 (CH_3, heptynyl-7), 19.0 (CH_2, heptynyl-3), 22.3 (CH_2, heptynyl-6), 23.8 (CH_3, C-9), 24.2 (CH_2, C-5), 24.3 (CH_3, C-10), 28.4 (CH_2, heptynyl-4), 28.6 (CH_2, C-6), 31.4 (CH_2, heptynyl-5), 49.2 (C_q, C-7), 51.6 (CH_2, C-8), 56.7 (CH, C-4), 62.8 (C_q, C-1), 73.1 (C_q, C-2), 82.2 (C_q, C-3), 80.7, 88.0, 89.7 and 90.2 (C_q, C≡C), 122,8 (C_q, Ph), 128.4 (CH, *m*-Ph), 128.8 (CH, *p*-Ph), 131.9 (CH, *o*-Ph).

(3a*S*,7*R*,7a*S*)-8,8-Dimethyl-7a-heptynyl-7-hydroxy-7-phenylethynyl-1,4,5,6,7,7a-hexahydro-3*H*-3a,6-methano-2,1-benzisothiazole 2,2-dioxide (2-endo-heptynyl-3-endo-phenylethinyl-3-exo-hydroxy-camphorsultam) (21b)

This compound was prepared in analogy to **21a** from **12b** and phenylethynyl lithium as starting material.

Yield 73%. Accurate EI-MS, m/z: Calcd for $C_{25}H_{32}NO_3S$ $[M + H]^+$: 426.2097. Found: 426.2095 (Δ [mmu] -0.2). EI-MS, *m/z*: 426 $[M + H]^+$, 408 $[M - H_2O]^+$, 362 $[M - SO_2]^+$, 345 $[M - H_2O - SO_2]^+$. IR spectrum (selected bands), cm^{-1}: 3414 ν(OH), 3322 s ν(NH), 2235 w ν(C≡C), 1330 and 1128 s $\nu(SO_2)$. ^{1}H NMR spectrum (500 MHz) in $CDCl_3$, δ

(ppm): 0.78 (t, 7.3 Hz, 3H, heptynyl-7), 1.02 (s, 3H, H-10), 1.17 (m, 2H, heptynyl-6), 1.28 (m, 2H, heptynyl-5), 1.48 (s, 3H, H-9), 1.54 (m, 2H, heptynyl-4), 1.76 (td, J = 12.2 Hz, J = 5.0 Hz, 1H, H-6 exo), 1.89 (m, 1H, H-5 exo), 2.04 (ddd, J = 13.8 Hz, J = 9.2 Hz, J = 4.8 Hz, 1H, H-5 endo), 2.18 (d, J = 4.9 Hz, 1H, H-4), 2.22 (m, 1H, H-6 endo), 2.27 (t, J = 7.2 Hz, 2H, heptynyl-3), 3.16 (s, br., 1H, OH), 3.29 (d, J = 13.7 Hz, 1H, H-8 syn), 3.33 (d, J = 13.7 Hz, 1H, H-8 anti), 5.08 (s, 1H, NH), 7.31 (m, 3H, *m*- and *p*-Ph), 7.45 (m, 2H, *o*-Ph). ^{13}C NMR spectrum (125 MHz) in $CDCl_3$, δ (ppm): 14.1 (CH_3, heptynyl-7), 19.2 (CH_2, heptynyl-3), 22.3 (CH_2, heptynyl-6), 23.9 (CH_3, C-9), 24.1 (CH_2, C-5), 24.3 (CH_3, C-10), 28.4 (CH_2, heptynyl-4), 28.6 (CH_2, C-6), 31.3 (CH_2, heptynyl-5), 49.1 (C_q, C-7), 51.5 (CH_2, C-8), 56.7 (CH, C-4), 62.2 (C_q, C-1), 72.9 (C_q, C-2), 78.4 (C_q, heptynyl-1), 82.4 (C_q, C-3), 88.1 (C_q) and 89.9 (C_q)(PhC≡C), 91.8 (C_q, heptynyl-2), 122,5 (C_q, Ph), 128.6 (CH, *m*-Ph), 128.9 (CH, *p*-Ph), 131.9 (CH, *o*-Ph).

Platinum-catalysed cycloisomerisations:

A solution of **21a** (150mg, 0.35 mmol) and $PtCl_2(PhCN)_2$ (8 mg, 0.017 mmol, 5 mol %) in $CHCl_3$ (2 mL) was heated to 60 °C overnight. Alternatively, the reaction mixture was heated under microwave irradiation to 80 °C for 30 min in a sealed vial. The initial pale yellow colour of the solution changed to orange-brownish. The solvent was evaporated and the residue was purified by column chromatography on SiO_2 (eluent CH_2Cl_2/Et_2O, gradient ratios 10:0, 10:1, 10:2, 10:5).

(2*S*,3a*S*)-10-Benzoyl-11,11-dimethyl-9-pentyl-4,5,6,7-tetrahydro-1*H*,3*H*-3a,6-methanocyclonona[2,1-*c*]isothiazol-7-one 2-oxide (22a)

Yield 67%. Elemental analysis calculated for $C_{25}H_{31}NO_3S$: C 70.55; H 7.34; N 3.29; found: C 70.10; H 7.19; N 3.26. Accurate EI-MS, m/z: Calcd for $C_{25}H_{32}NO_3S$ $[M + H]^+$: 426.2097. Found: 426.2091 (Δ [mmu] –0.6). EI-MS, *m/z*: 426 $[M + H]^+$, 410 $[M - NH_3]^+$, 377 $[M - SO]^+$. IR spectrum (selected bands), cm^{-1}: 3110 w ν(N-H····O=), 1680 and 1611 s ν(C=O), 1533 s ν(C=C), 1098 m ν(SO). ^{1}H NMR spectrum (500 MHz) in $CDCl_3$, δ

(ppm): 0.76 (t, J = 7.1 Hz, 3H, pentyl-5), 0.99 (m. 2H, pentyl-3), 1.08 (m, 2H, pentyl-4), 1.30 (m, 2H, pentyl-2), 1.32 (s, 3H, H-10), 1.35 (s, 3H, H-9), 1.70 (m, 1H, pentyl-1), 1.76 (m, 1H, pentyl-1), 1.97 (m, 1H, H-5 endo), 2.15 (m, 1H, H-6 exo), 2.22 (m, 1H, H-5 exo), 2.62 (d, J = 6.1 Hz, 1H, H-4), 2.74 (m, 1H, H-6 endo), 3.08 (d, J = 14.1Hz, 1H, H-8 anti), 3.50 (d, J = 14.1 Hz, 1H, H-8 syn), 5.94 (s, 1H, =CH), 7.38 (t, J = 7.2 Hz, 2H, *m*-Ph), 7.45 (t, J = 7.4Hz, 1H, *p*-Ph), 7.65 (d, J = 7.8 Hz, 2H, *o*-Ph), 11.97 (s, 1H, NH). ^{13}C NMR spectrum (125 MHz) in $CDCl_3$, δ (ppm): 14.1 (CH_3, pentyl-5), 22.0 (CH_3, C-9), 22.4 (CH_2, pentyl-4), 25.4 (CH_2, C-5), 27.6 (CH_2, pentyl-2), 28.9 (CH_3, C-10), 31.5 (CH_2, pentyl-3), 35.6 (CH_2, C-6), 39.4 (CH_2, pentyl-1), 47.1 (C_q, C-7), 60.3 (CH_2, C-8), 61.5 (C_q, C-1), 66.8 (CH, C-4), 109.0 (C_q, C2–*C*=), 128.27 (CH) and 128.28 (CH)(*o*- and *m*-Ph), 129.3 (CH, =CH), 131.8 (CH, *p*-Ph), 138.4 (C_q, HC=*C*), 140.5 (C_q, Ph), 163.4 (C_q, C-2), 198.3 (C_q, Ph-C=O), 210.8 (C_q, C-3).

(2*S*,3a*S*)-11,11-Dimethyl-10-(1-oxohexyl)-9-phenyl-4,5,6,7-tetrahydro-1*H*,3*H*-3a,6-methanocyclonona[2,1-*c*]isothiazol-7-one 2-oxide (22b)

This compound was prepared in analogy to **22a** from **21b** as starting material. The reaction provided **22b** and **23** as a 1:1 mixture. These products were separated by column chromatography on SiO_2 (eluent ethyl acetate/hexane 1:1).

Yield 37%. Elemental analysis calculated for $C_{25}H_{31}NO_3S$: C 70.55; H 7.34; N 3.29; found: C 70.29; H 7.24; N 3.23. Accurate EI-MS, m/z: Calcd for $C_{25}H_{32}NO_3S$ $[M + H]^+$: 426.2093. Found: 426.2091 (Δ [mmu] –0.4). EI-MS, *m/z*: 426 $[M + H]^+$, 377 $[M – SO]^+$. R_f = 0.35 (eluent ethyl acetate/hexane 1:1). IR spectrum (selected bands), cm^{-1}: 3105 w ν(N-H····O=), 1695 and 1623 s ν(C=O), 1533 s ν(C=C), 1100 m ν(SO). ^{1}H NMR spectrum (500 MHz) in $CDCl_3$, δ (ppm): 0.75 (t, J = 7.2 Hz, 3H, pentyl-5), 0.96 (m. 2H, pentyl-3), 1.10 (m, 2H, pentyl-4), 1.13 (m, 1H, pentyl-2), 1.27 (s, 3H, H-9), 1.30 (s, 3H, H-10), 1.40 (m, 1H, pentyl-2), 1.89 (ddd, J = 15.8 Hz, J = 8.6 Hz, J = 6.0 Hz, 1H, pentyl-1), 2.28 (ddd, J = 15.8 Hz, J = 8.6 Hz, J = 6.1 Hz, 1H, pentyl-1), 1.97 (m, 1H, H-5 endo),

2.08 (m, 1H, H-6 exo), 2.18 (m, 1H, H-5 exo), 2.61 (d, J = 5.9 Hz, 1H, H-4), 2.64 (m, 1H, H-6 endo), 3.09 (d, J = 14.2 Hz, 1H, H-8 anti), 3.47 (d, J = 14.2 Hz, 1H, H-8 syn), 6.60 (s, 1H, =CH), 7.34 (m, 5H, Ph), 12.76 (s, 1H, NH). ^{13}C NMR spectrum (125 MHz) in $CDCl_3$, δ (ppm): 14.1 (CH_3, pentyl-5), 21.9 (CH_3, C-9), 22.3 (CH_2, pentyl-4), 25.4 (CH_2, C-5), 24.8 (CH_2, pentyl-2), 28.6 (CH_3, C-10), 31.2 (CH_2, pentyl-3), 34.8 (CH_2, C-6), 41.7 (CH_2, pentyl-1), 47.6 (C_q, C-7), 59.5 (CH_2, C-8), 61.5 (C_q, C-1), 66.7 (CH, C-4), 107.5 (C_q, C2–<u>*C*</u>=), 126.6 (CH) and 129.4 (CH)(*o*- and *m*-Ph), 128.9 (CH, *p*-Ph), 131.4 (CH, =CH), 136.9 (C_q, HC=<u>*C*</u>), 141.5 (C_q, Ph), 163.6 (C_q, C-2), 204.9 (C_q, pentyl-C=O), 209.4 (C_q, C-3).

(3*R*,4a*S*,7*S*,7a*R*,9b*S*)-5,6,7,7a-Tetrahydro-7a-hydroxy-11,11-dimethyl-1-pentyl-9-phenyl-4*H*-4a,7-methanoindeno[7,1-*de*][1,2]oxathiepin-3,9b-imine 3-oxide (23)

Yield 37%. Accurate ESI-MS, m/z: Calcd for $C_{25}H_{31}NNaO_3S$ $[M + Na]^+$: 448.1915. Found: 448.1917 (Δ [mmu] 0.2). ESI-MS: (M = 425.595) 448 $[M + Na]^+$. R_f = 0.75 (eluent ethyl acetate/hexane 1:1). IR spectrum (selected bands), cm^{-1}: 3303 s ν(OH), 1653 m ν(C=C), 1324 and 1059 s ν(SON). ^{1}H NMR spectrum (500 MHz) in $CDCl_3$, δ (ppm): 0.76 (t, 7.1 Hz, 3H, pentyl-5), 0.95 (m. 2H, pentyl-3), 0.97 (s, 3H, H-9 or H-10), 1.08 (m, 2H, pentyl-4), 1.10 (m, 2H, pentyl-2), 1.43 (s, 3H, H-10 or H-9), 1.50 (m, 1H, H-5 endo), 1.72 (m, 1H, H-6 exo), 1.75 (m, 2H, pentyl-1), 1.78 (m, 1H, H-5 exo), 1.90 (m, 1H, H-6 endo), 2.11 (d, J = 5.8 Hz, 1H, H-4), 2.76 (s, 1H, OH), 3.07 (d, J = 14.0Hz, 1H, H-8 anti or syn), 3.43 (d, J = 14.0 Hz, 1H, H-8 syn or anti), 5.80 (s, 1H, =CH), 7.23 (m, 2H, *o*-Ph), 7.34 (m, 3H, *m*- and *p*-Ph). ^{13}C NMR spectrum (125 MHz) in $CDCl_3$, δ (ppm): 14.1 (CH_3, pentyl-5), 22.4 (CH_2, pentyl-4), 22.5 (CH_3, C-9 or C-10), 23.2 (CH_3, C-10 or C-9), 24.8 (CH_2, C-5), 26.7 (CH_2, pentyl-2), 27.7 (CH_2, C-6), 31.3 (CH_2, pentyl-3), 30.2 (CH_2, pentyl-1), 51.2 (C_q, C-7), 52.3 (CH_2, C-8), 53.2 (CH, C-4), 60.6 (C_q, C-1), 84.0 (C_q,C-2), 90.0 (C_q, C-3), 125.5 (C_q, C2-<u>*C*</u>=), 128.2 (2 CH, *o*- and *p*-Ph), 128.5 (CH, *m*-Ph), 136.0 (C_q, CH=<u>*C*</u>), 138.5 (CH, =CH), 140.1 (C_q, Ph), 151.4 (C_q, =C-O).

References

1. Glahsl, G.; Herrmann, R. *J. Chem. Soc., Perkin Trans. 1*, **1988**, 1753-1757.

2. Belsey, S.; Danks, T. N.; Wagner, G. *Synth. Commun*. **2006**, *36*, 1019-1024.

3. Verfürth, U.; Herrmann, R. *J. Chem. Soc., Perkin Trans. 1*, **1990**, 2919-2928.

4. Lampropoulou, M.; Herrmann, R.; Wagner, G. *Tetrahedron* **2004**, *60*, 4635-4643.

5. M. Čudić, Campher-Derivate als chirale Reagentien in stereoselektiven Reaktionen, Dissertation, Technical University of Munich, Germany, **1996**.

Copies of ^{1}H and ^{13}C NMR spectra

^{1}H NMR spectrum (500 MHz) of compound 12a

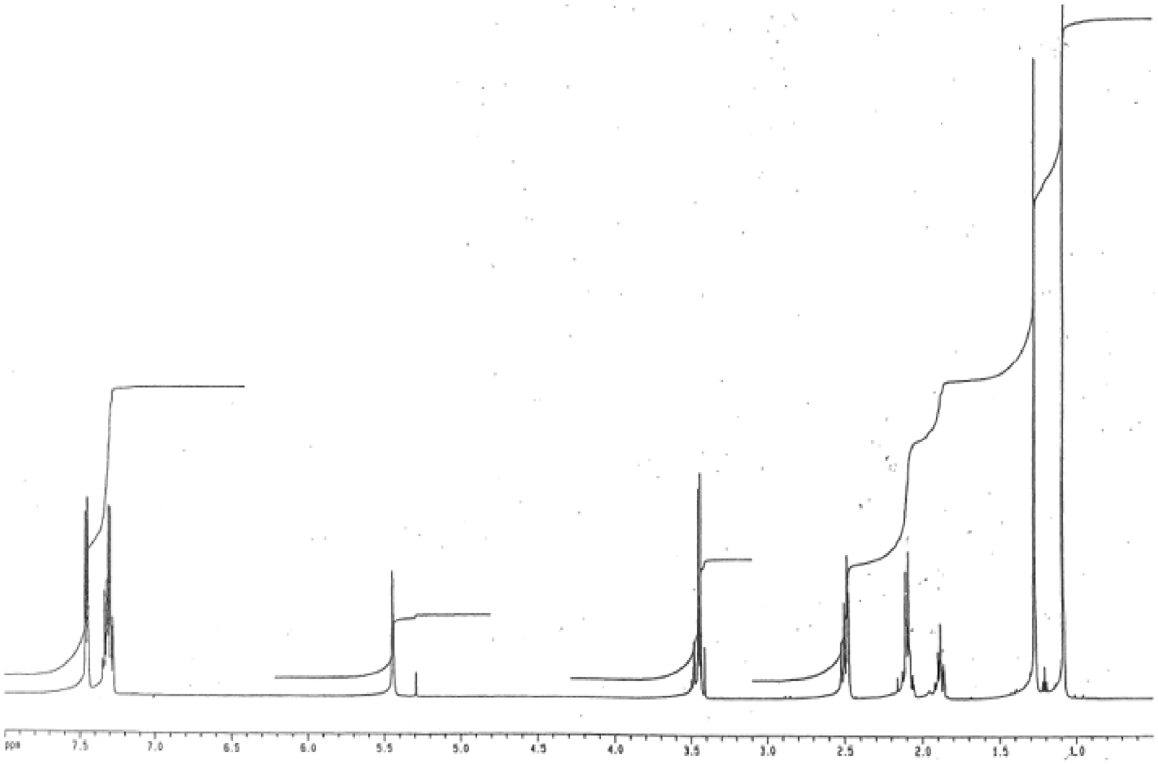

^{13}C NMR spectrum (125 MHz) of compound 12a

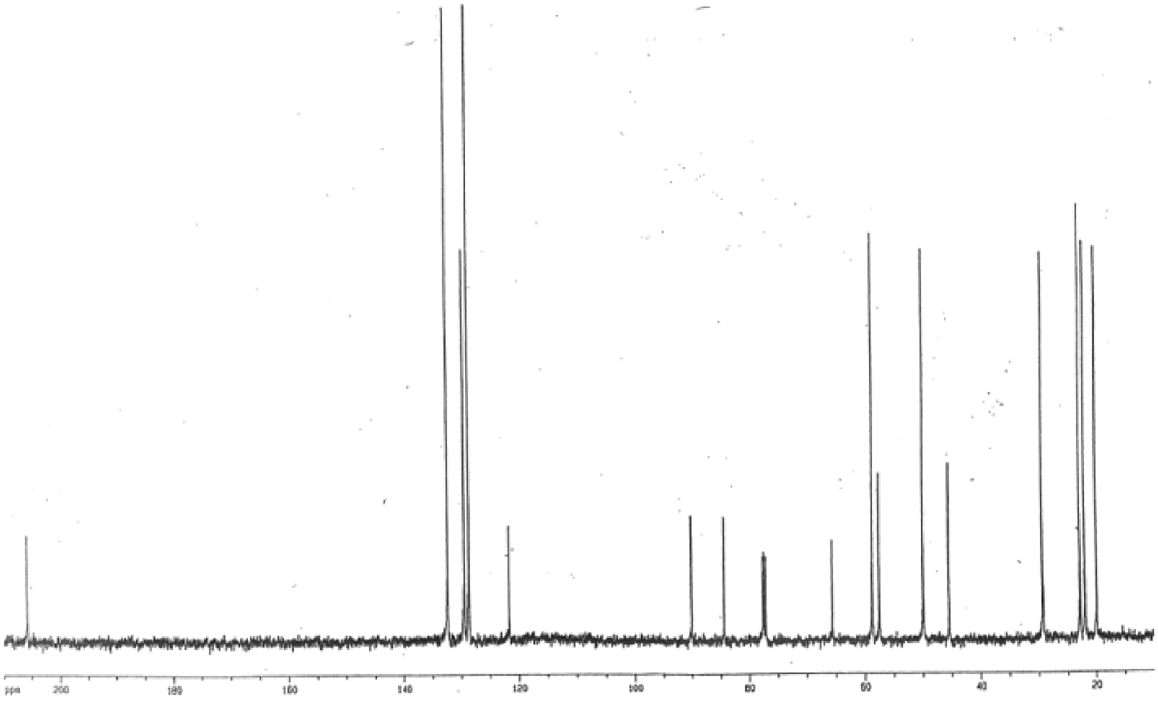

^{1}H NMR spectrum (500 MHz) of compound 13a

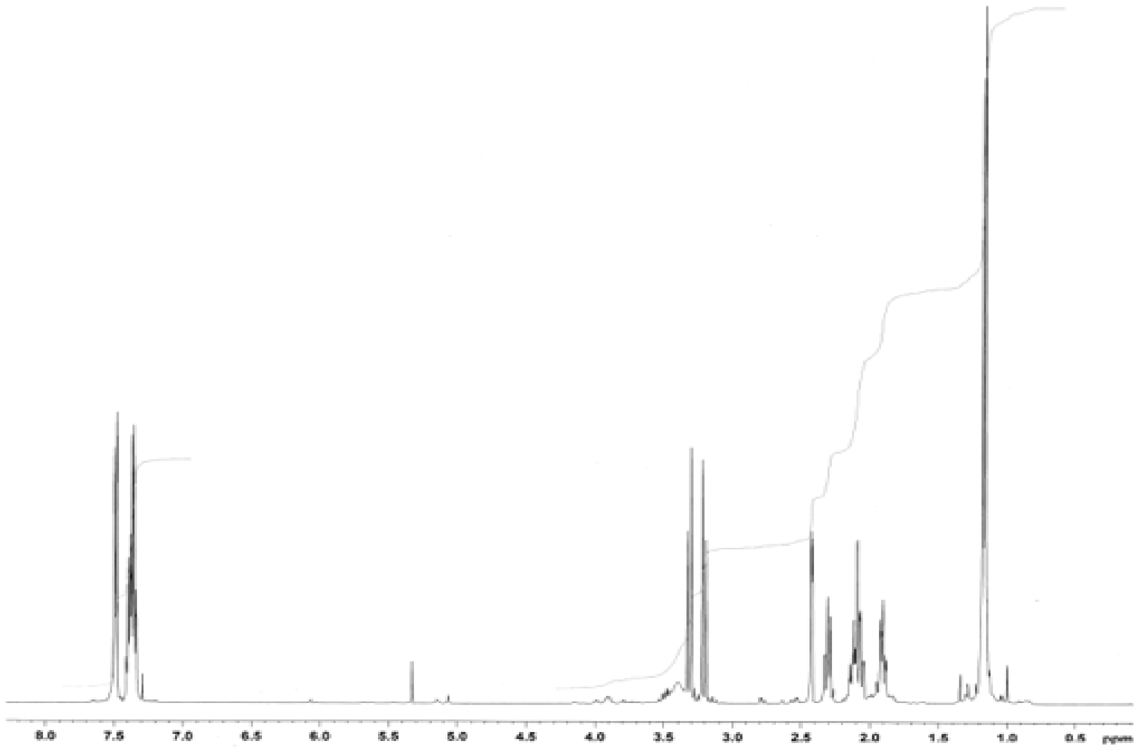

^{13}C NMR spectrum (125 MHz) of compound 13a

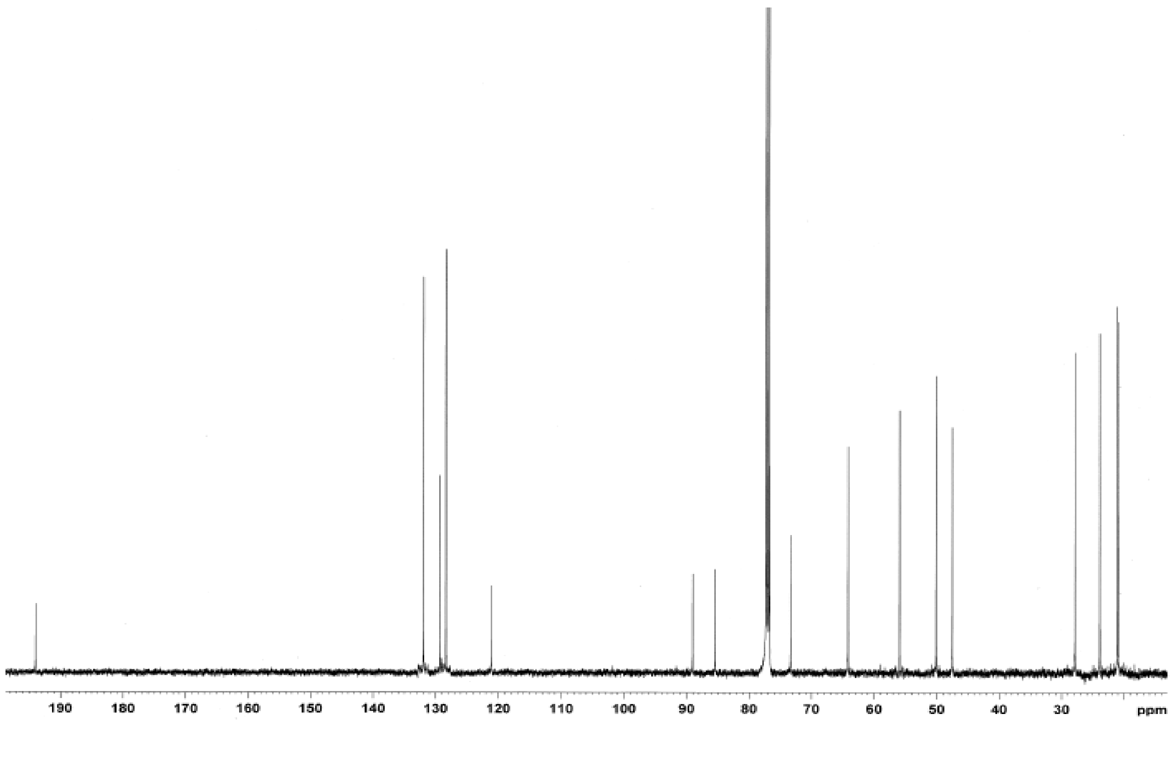

^{1}H NMR spectrum (500 MHz) of compound 12b

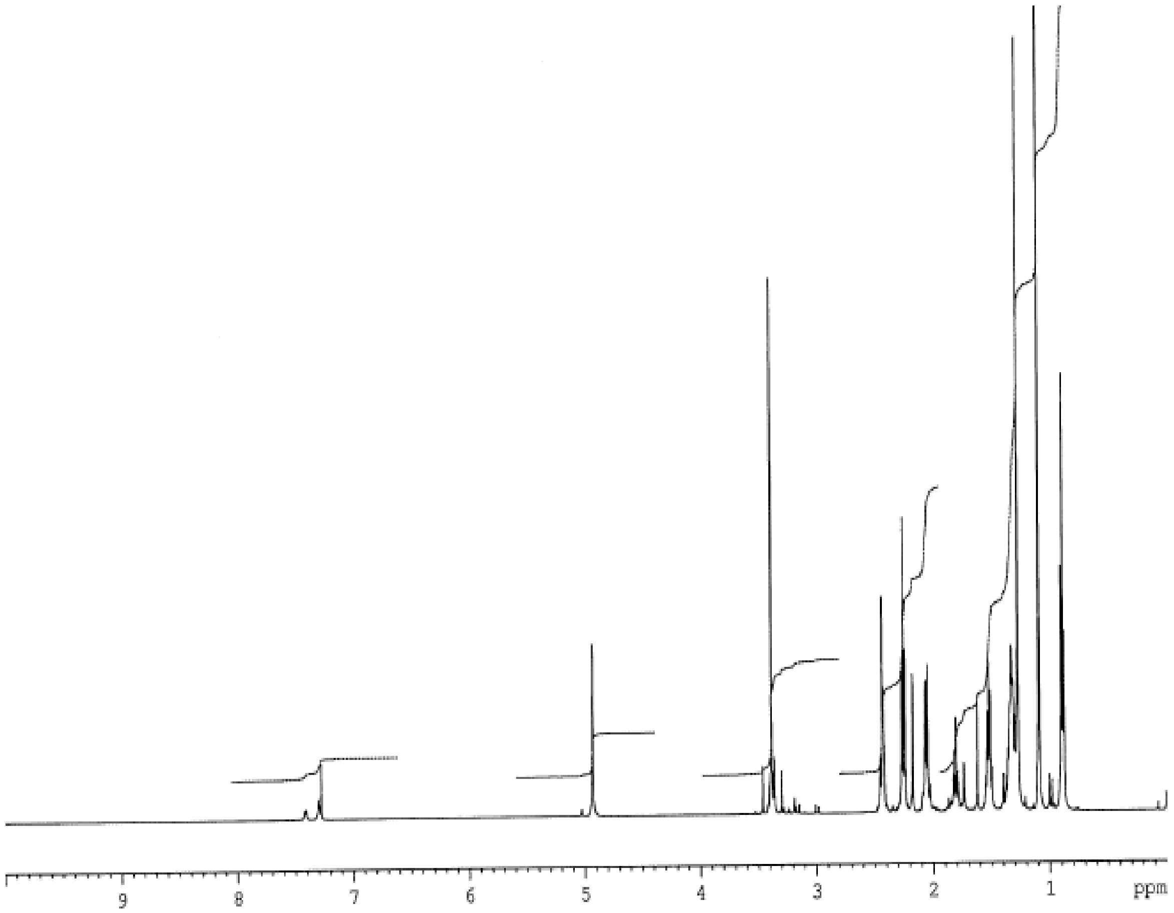

^{13}C NMR spectrum (125 MHz) of compound 12b

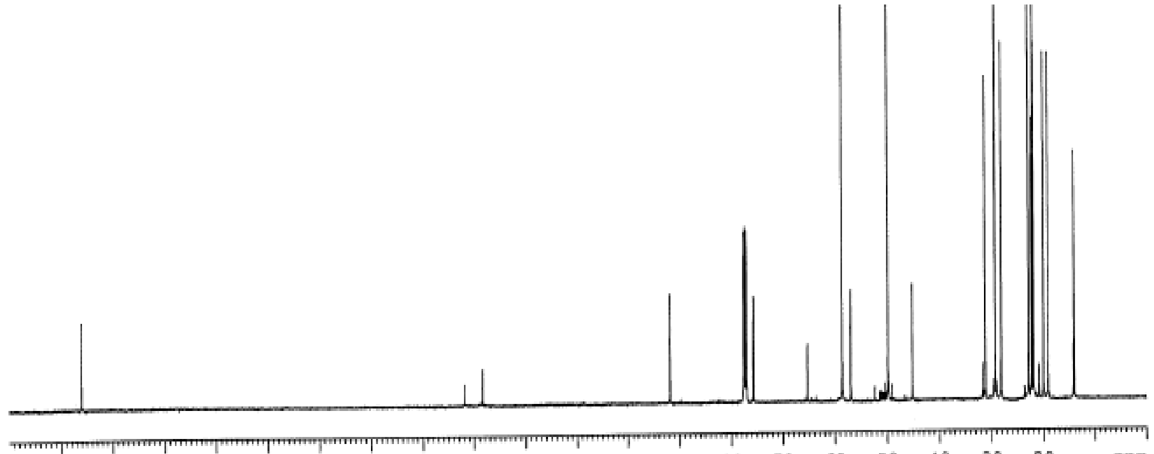

^{1}H NMR spectrum (500 MHz) of compound 17a

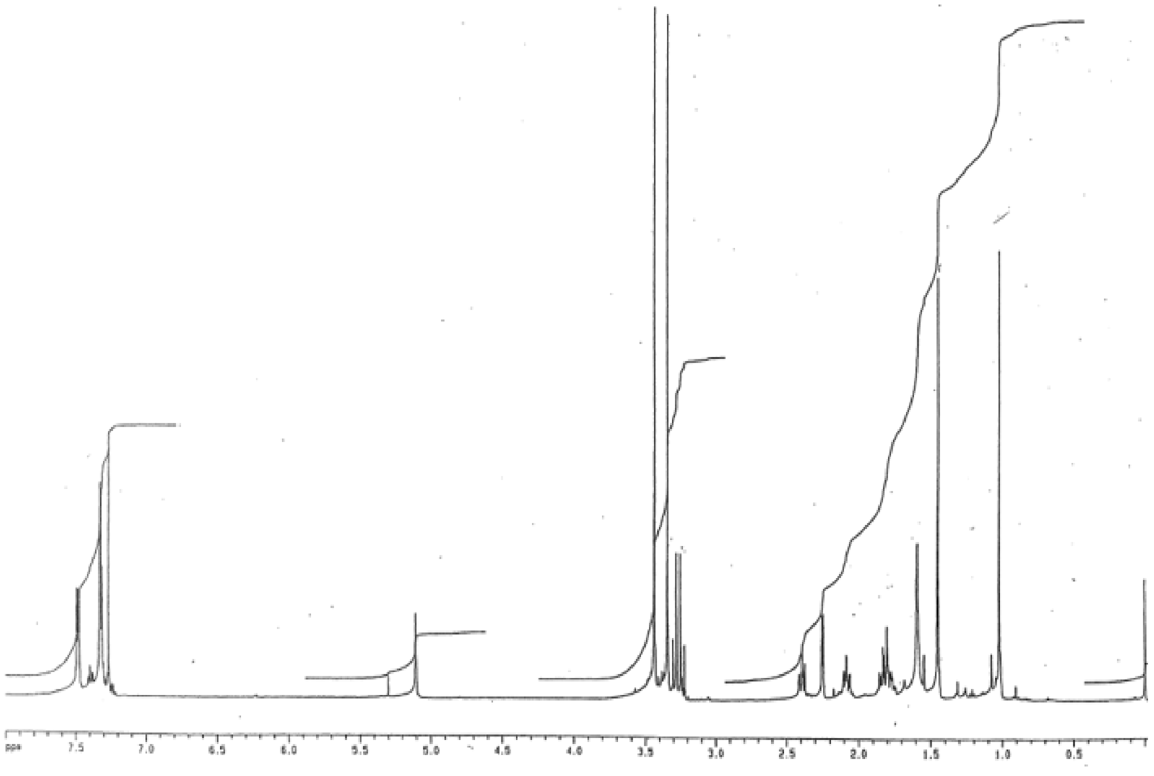

^{13}C NMR spectrum (125 MHz) of compound 17a

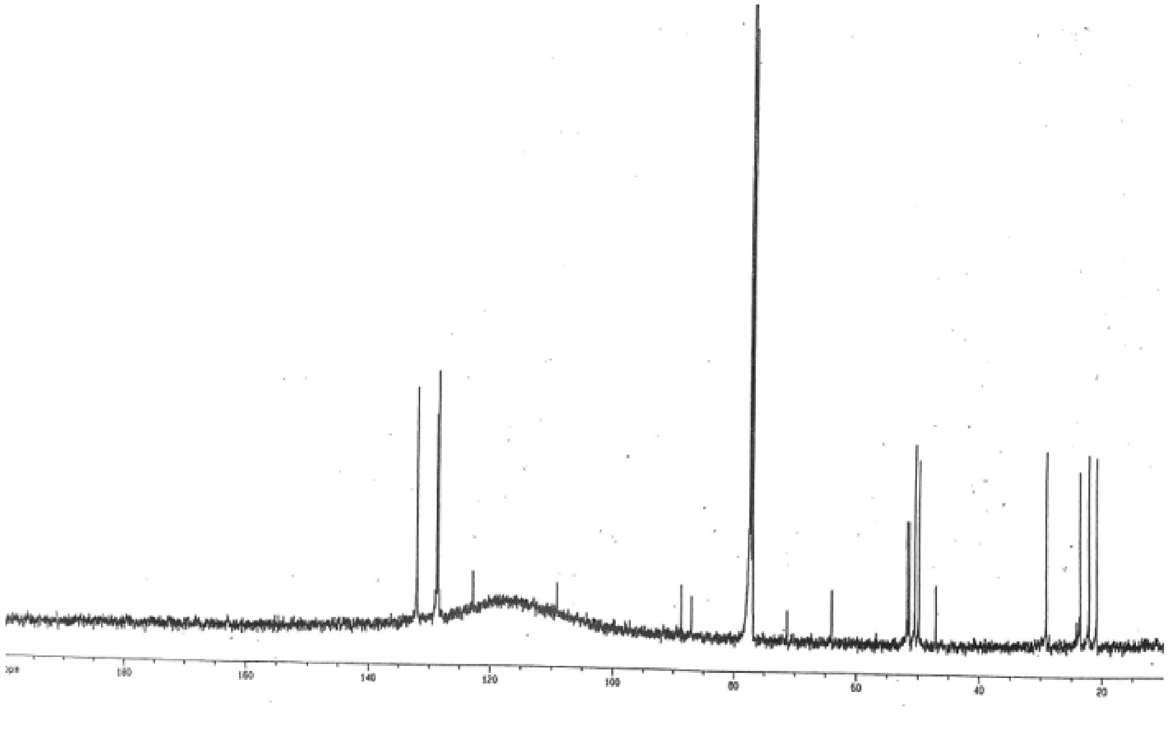

^{1}H NMR spectrum (500 MHz) of compound 17b

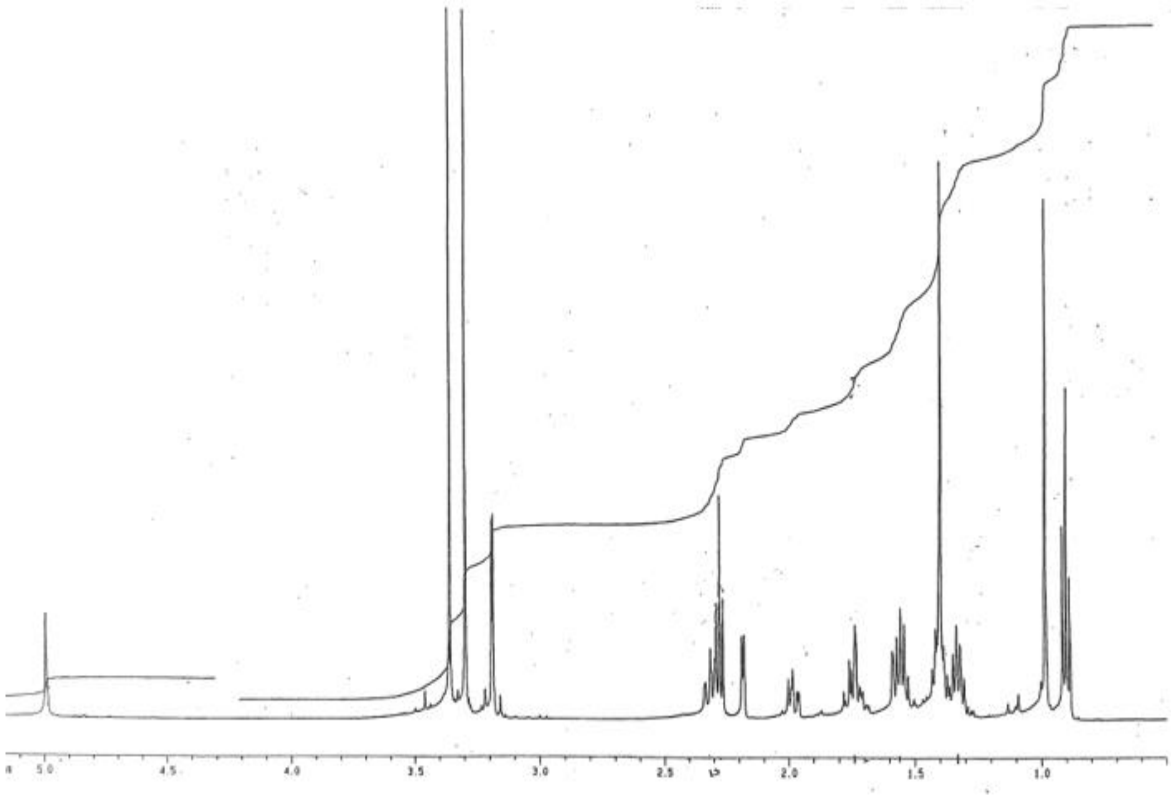

^{13}C NMR spectrum (125 MHz) of compound 17b

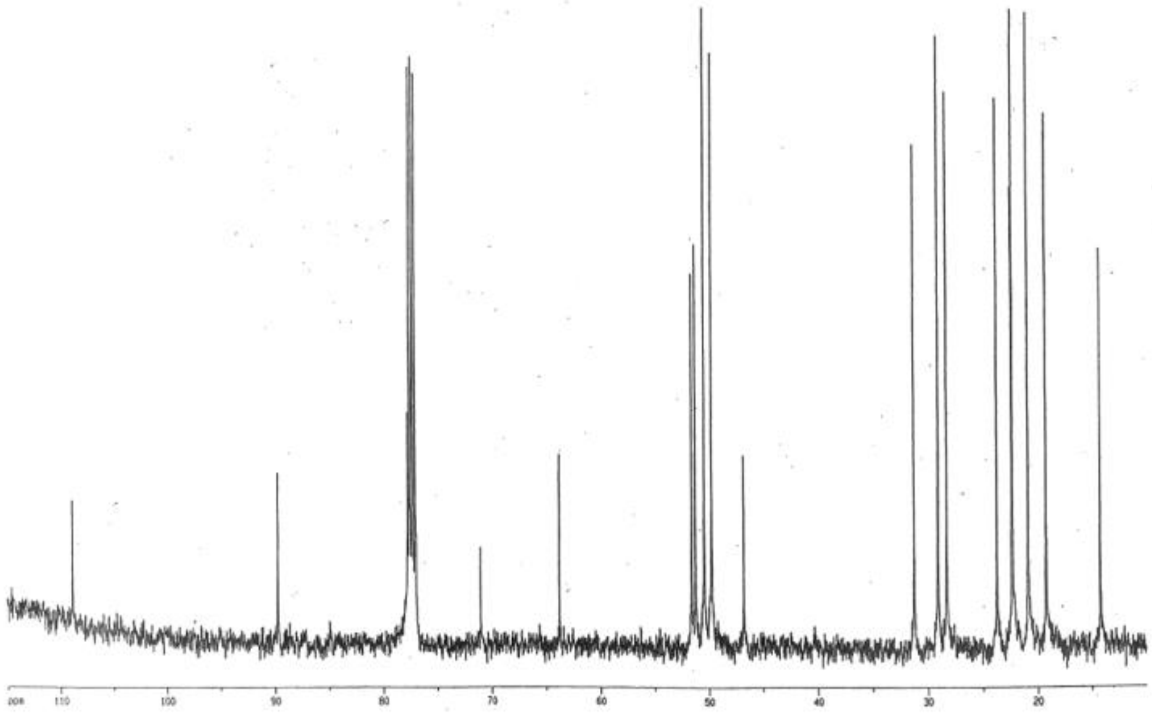

^{1}H NMR spectrum (500 MHz) of compound 18

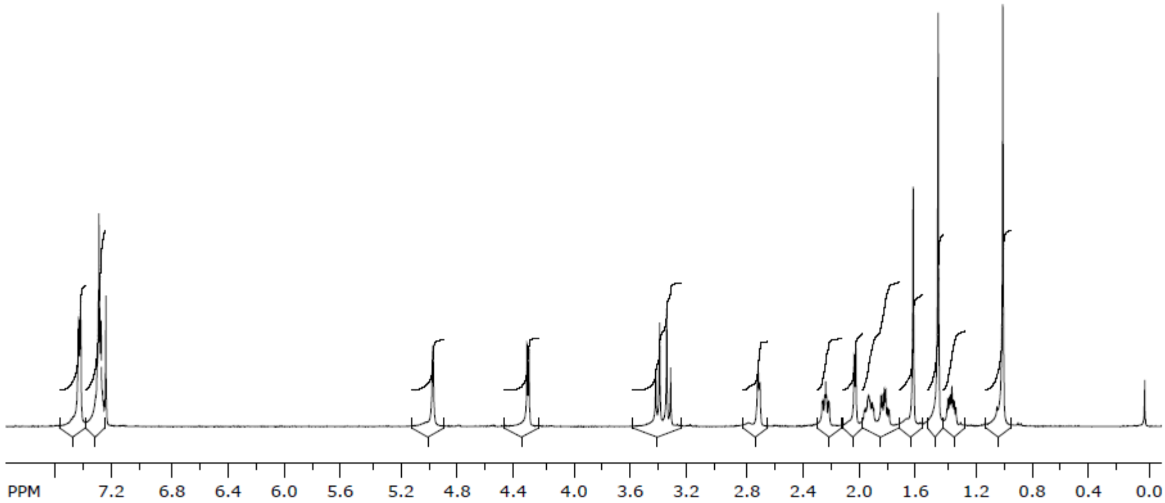

^{1}H NMR spectrum (500 MHz) of compound 19

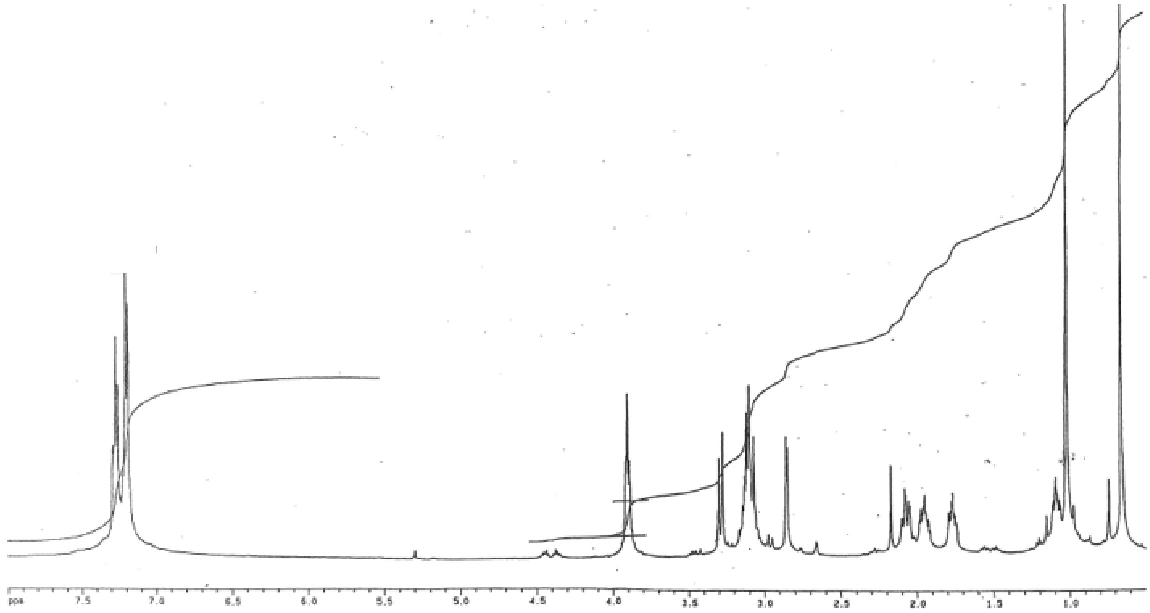

^{13}C NMR spectrum (125 MHz) of compound 19

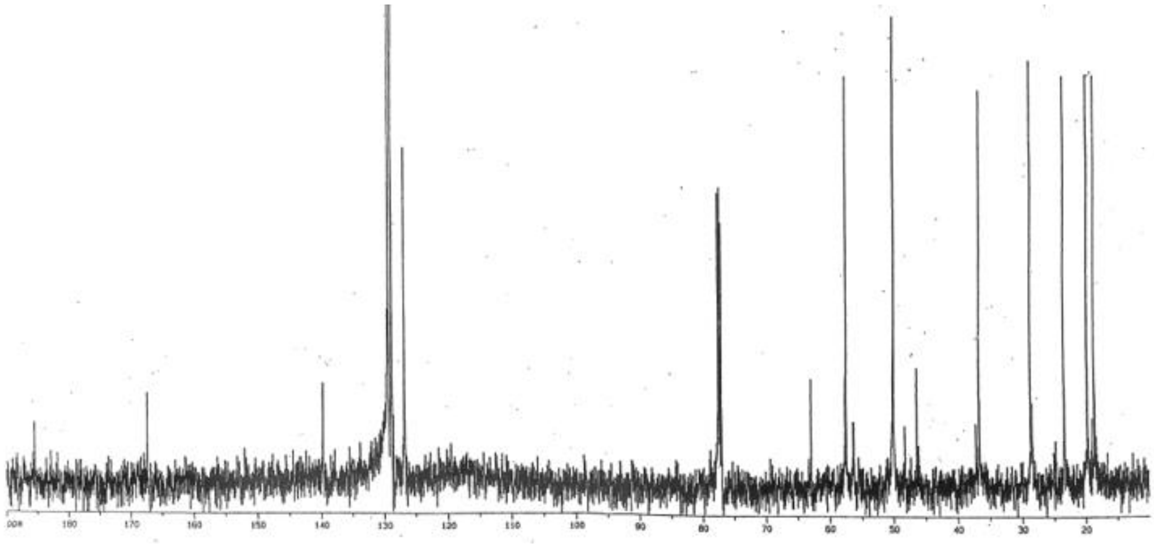

^{1}H NMR spectrum (500 MHz) of compound 20

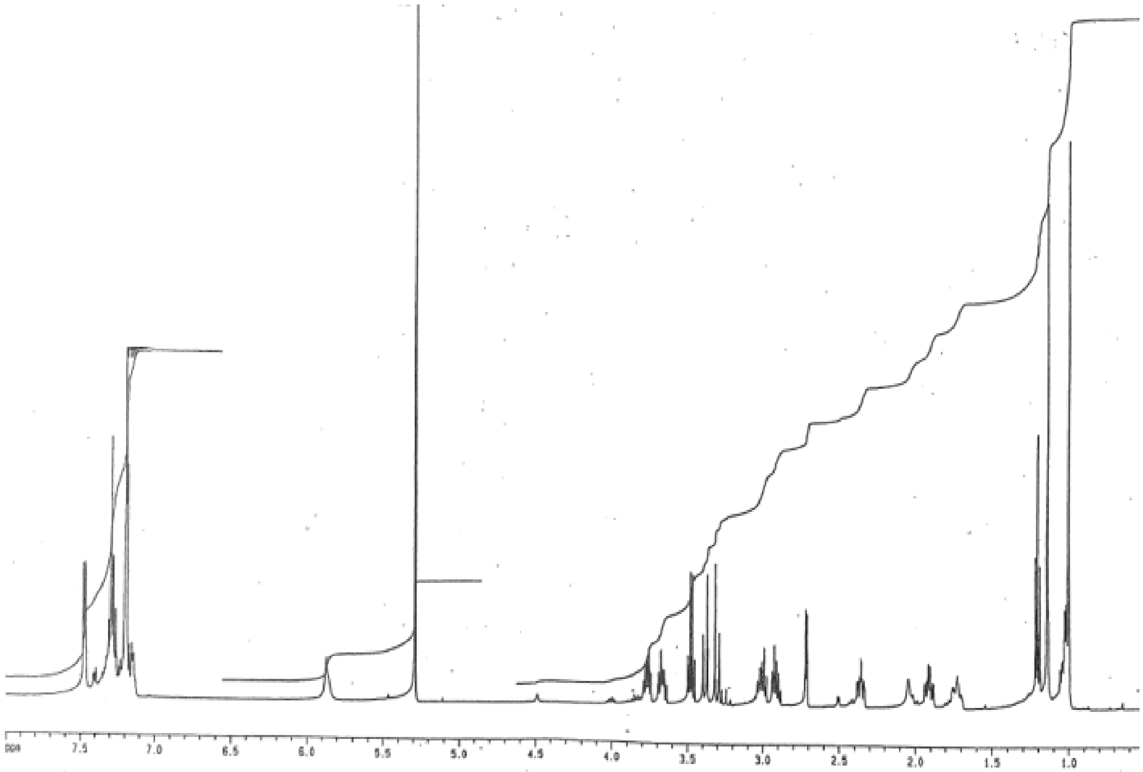

^{13}C NMR spectrum (125 MHz) of compound 20

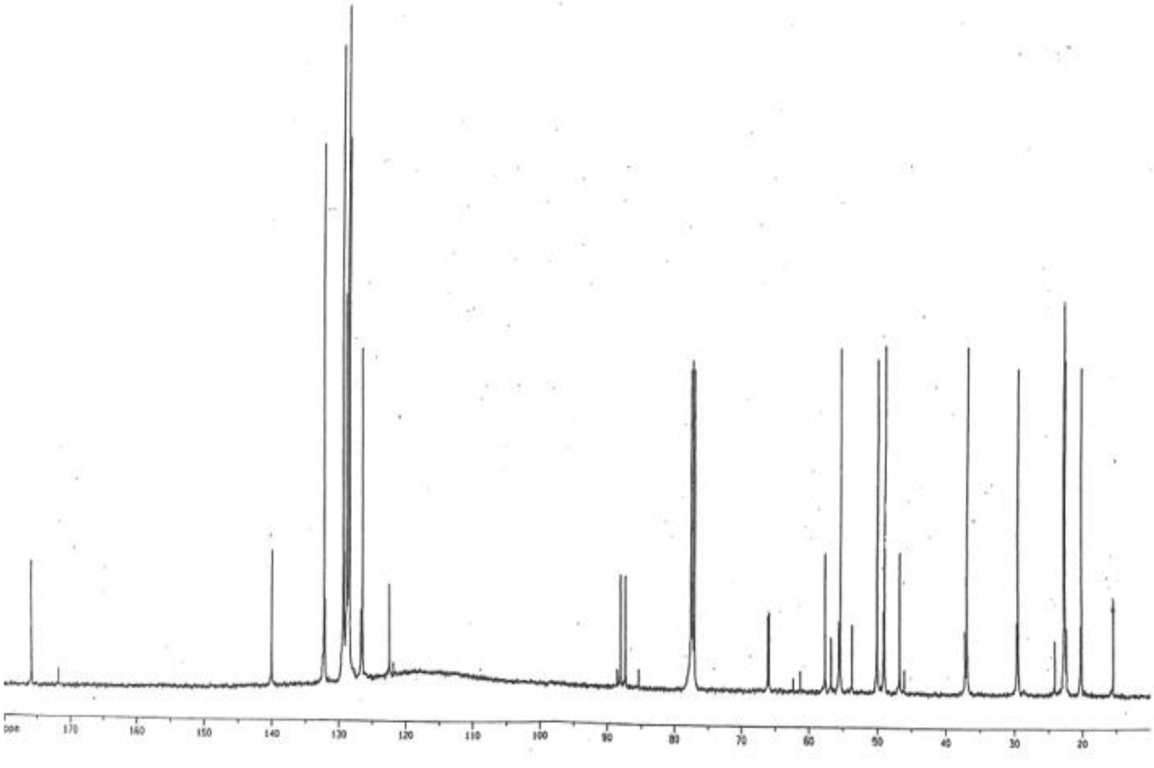

^{1}H NMR spectrum (500 MHz) of compound 21a

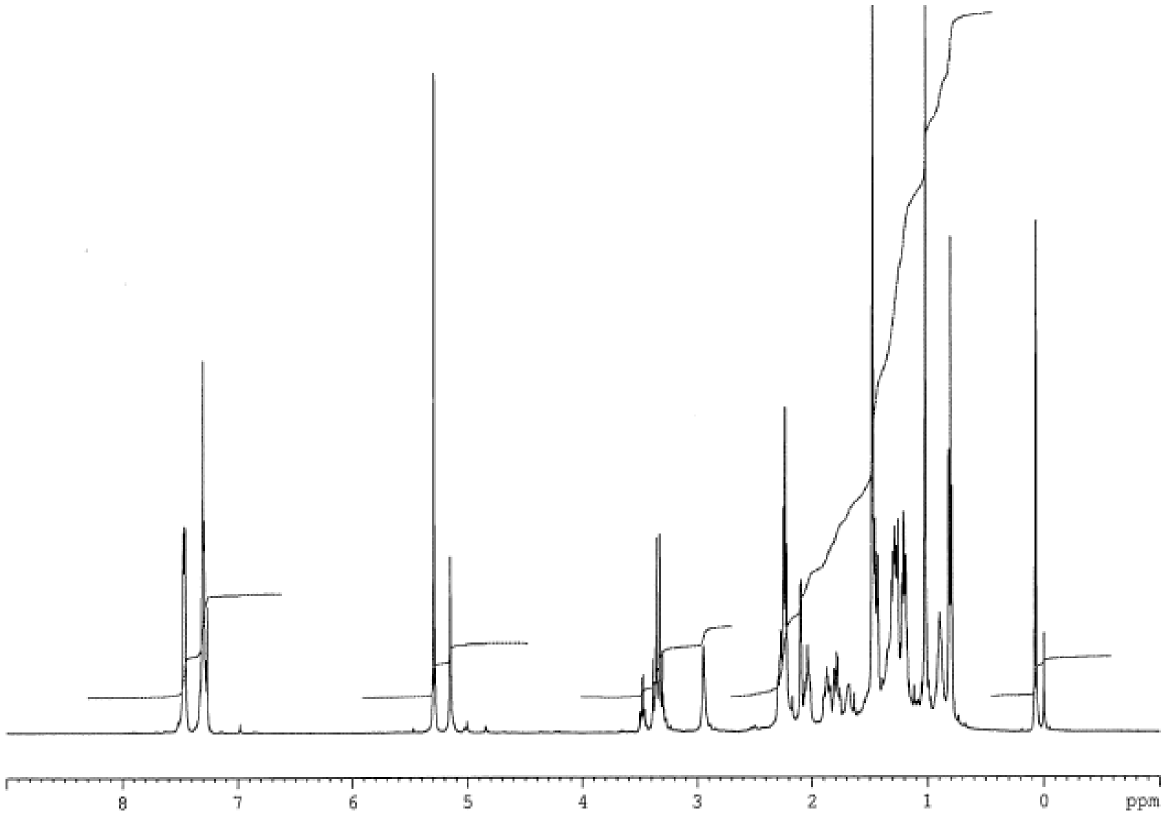

^{13}C NMR spectrum (125 MHz) of compound 21a

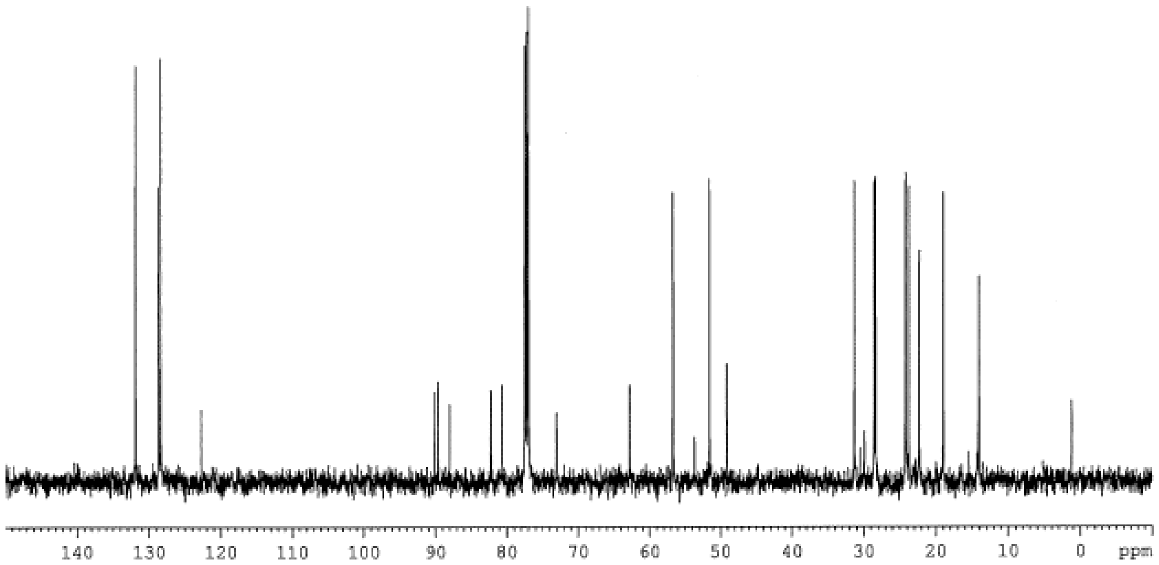

^{1}H NMR spectrum (500 MHz) of compound 21b

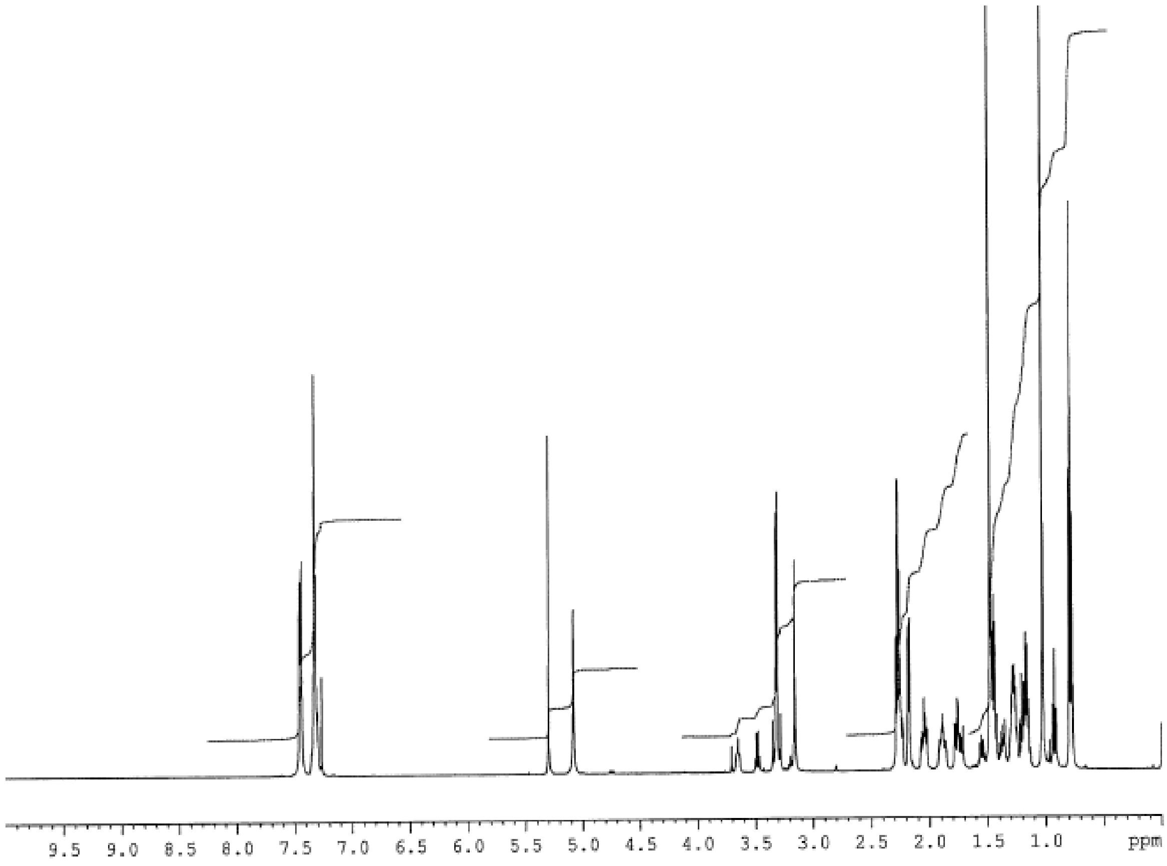

^{13}C NMR spectrum (125 MHz) of compound 21b

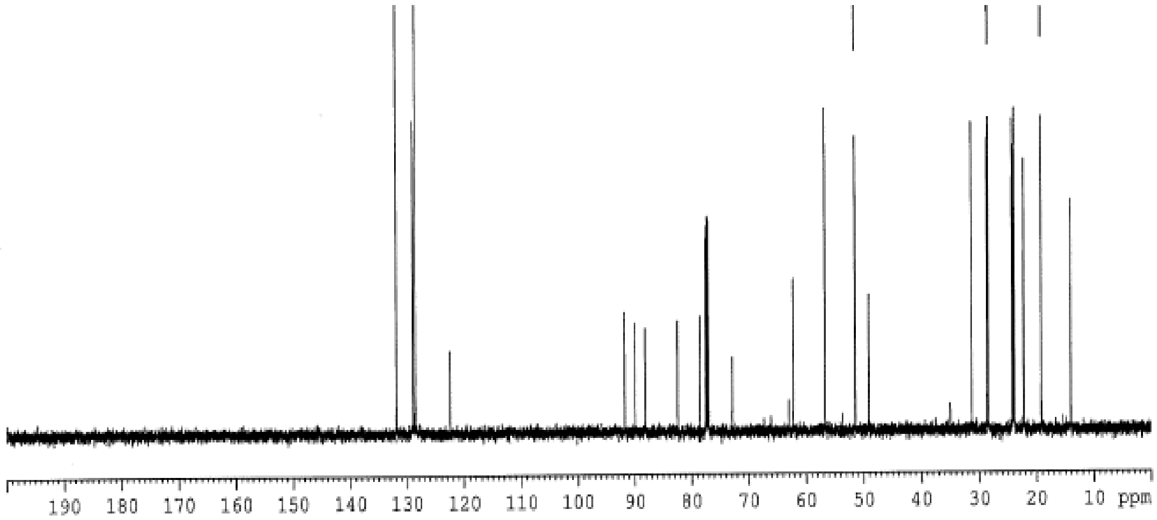

^{1}H NMR spectrum (500 MHz) of compound 22a

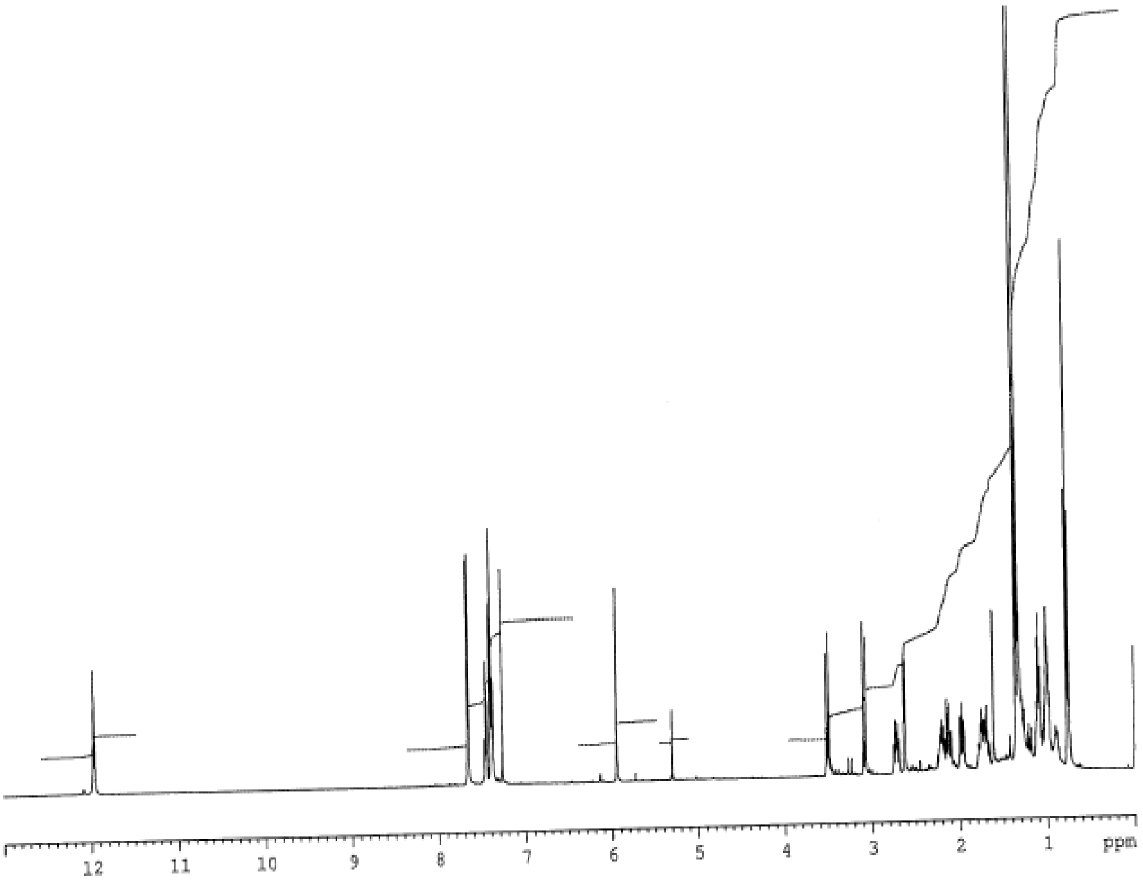

^{13}C NMR spectrum (125 MHz) of compound 22a

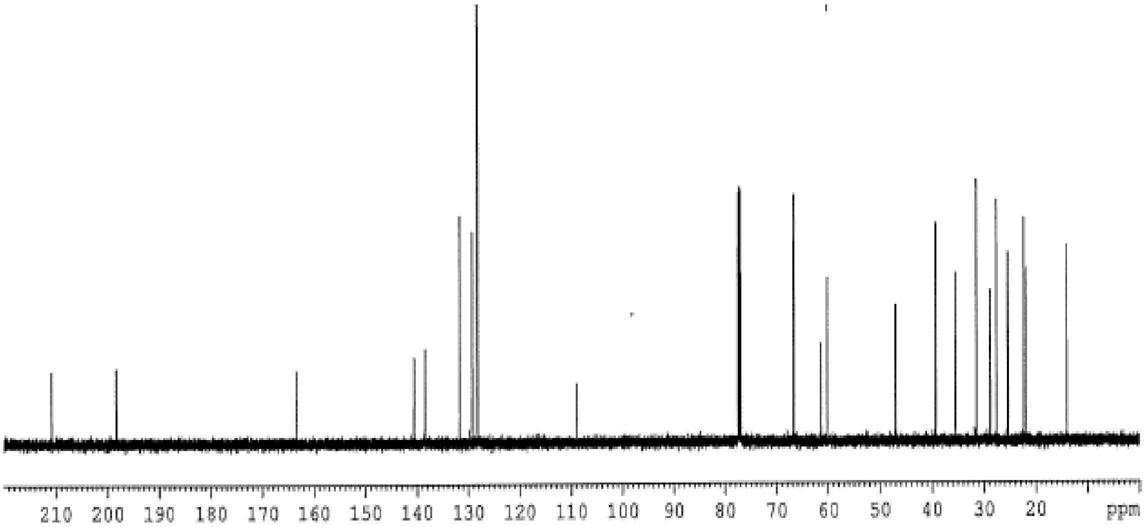

^{1}H NMR spectrum (500 MHz) of the crude reaction product 21b → 22b + 23

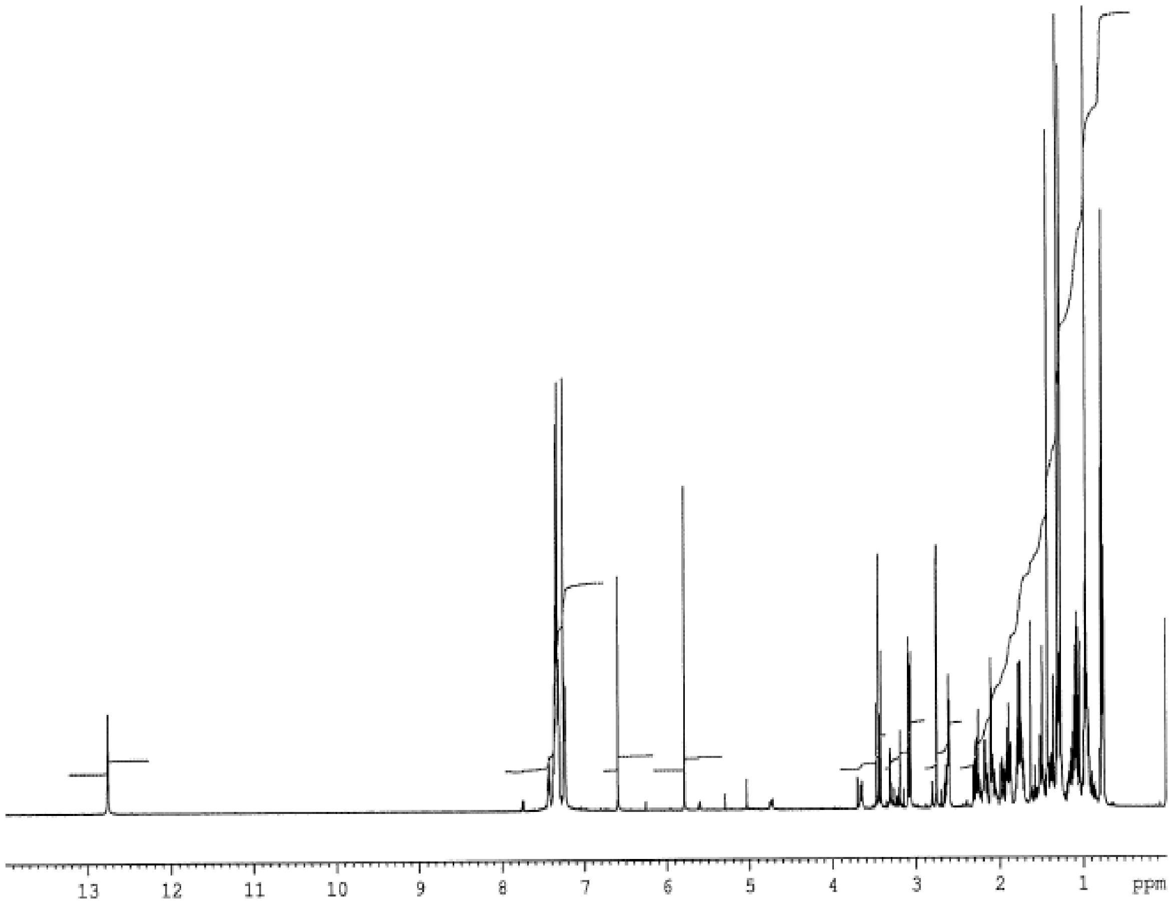

^{13}C NMR spectrum (125 MHz) of the crude reaction product 21b → 22b + 23

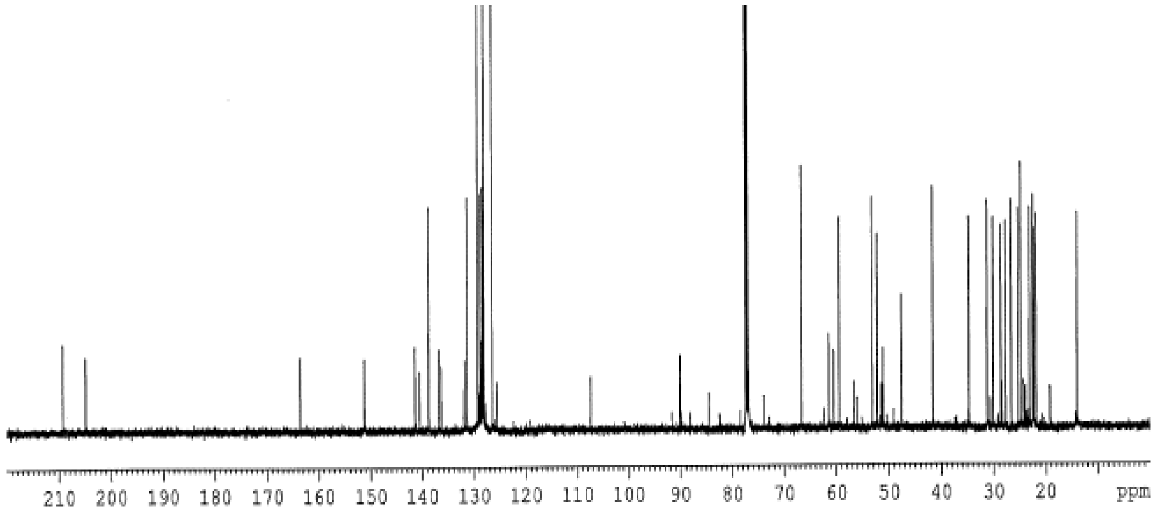

^{1}H NMR spectrum (500 MHz) of compound 22b

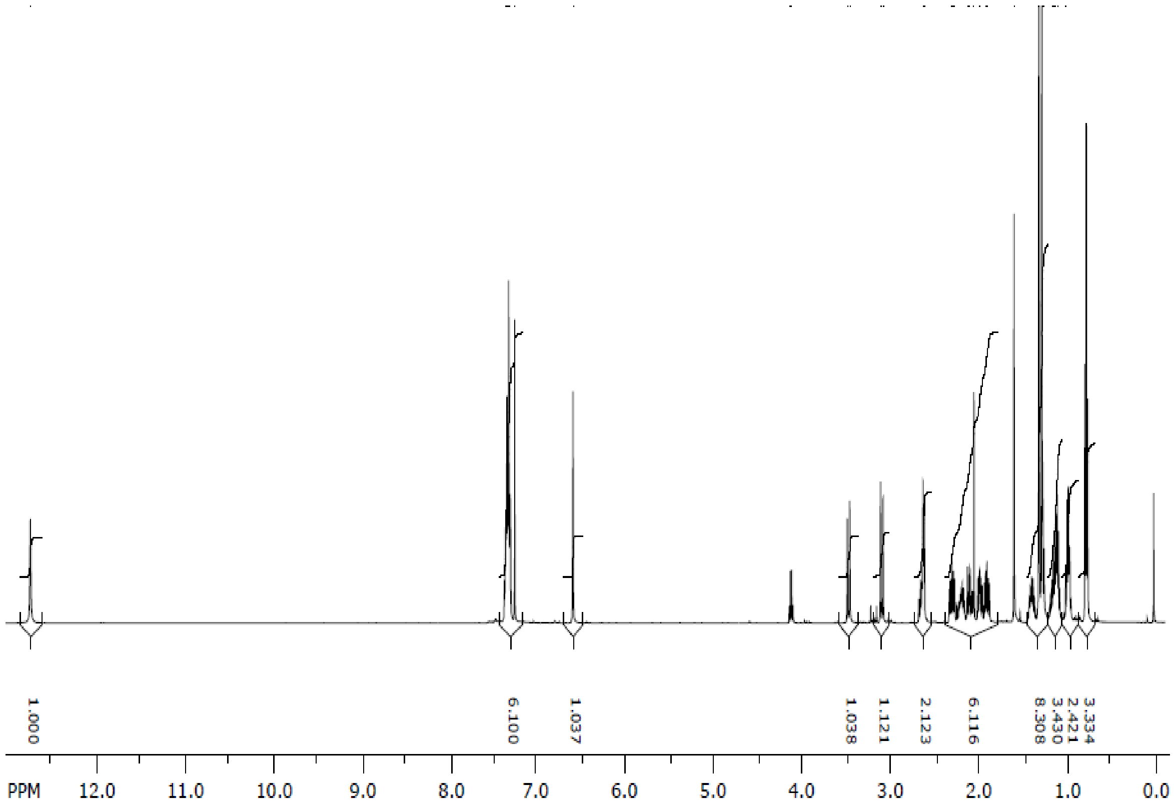

^{13}C NMR spectrum (125 MHz) of compound 22b

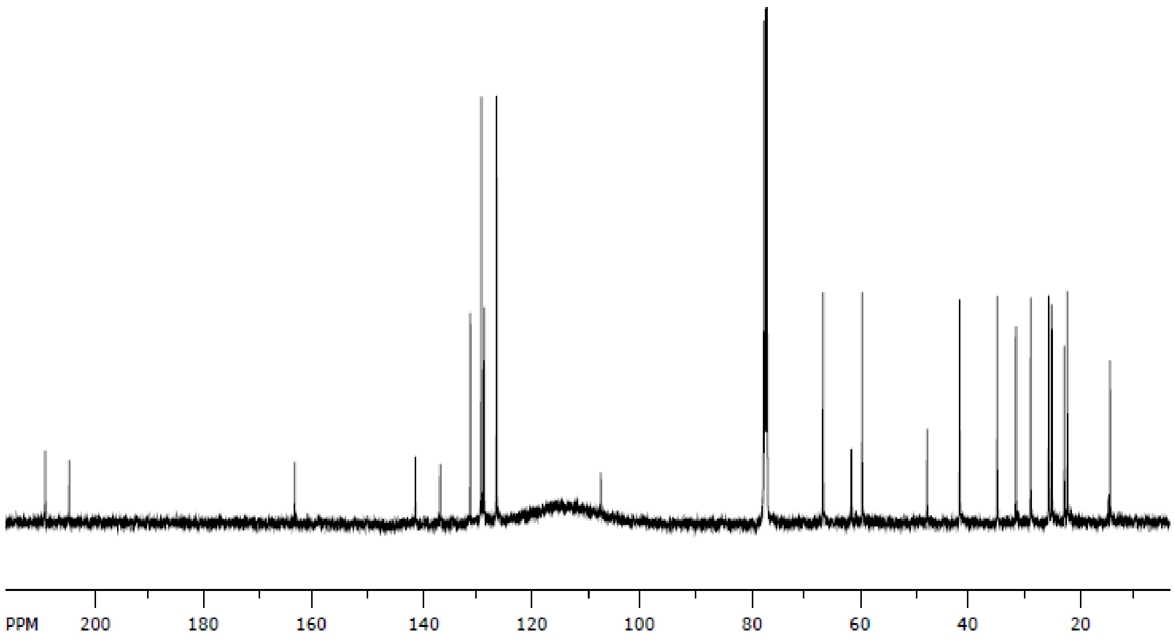

^{1}H NMR spectrum (500 MHz) of compound 23

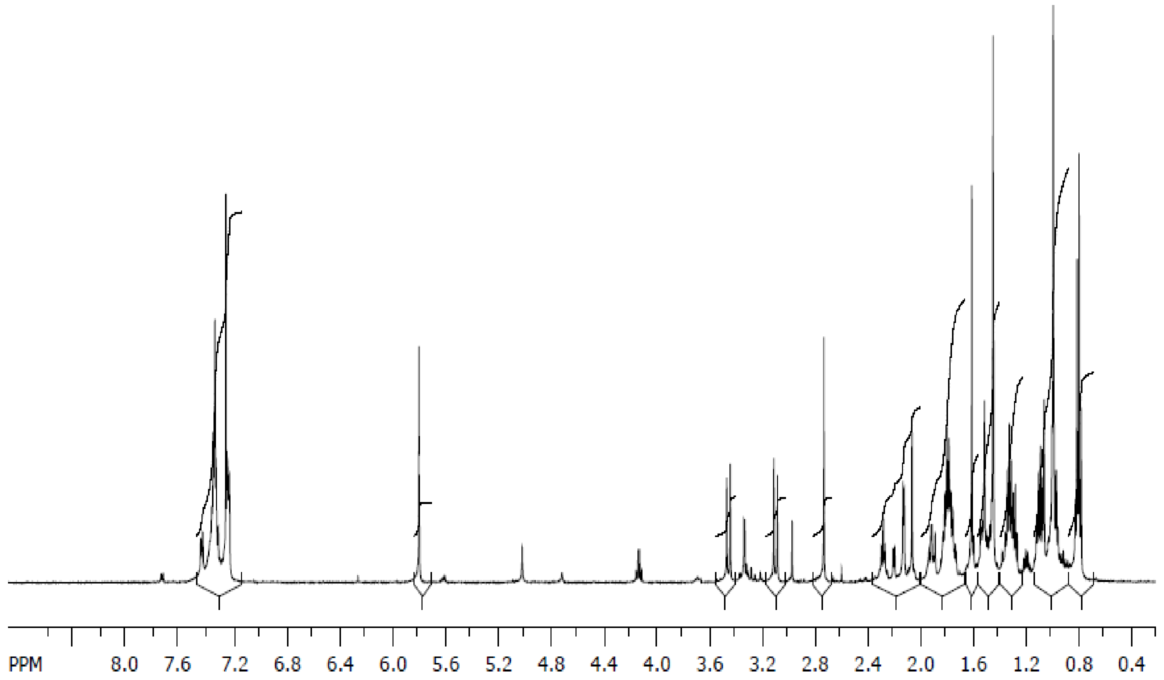

^{13}C NMR spectrum (125 MHz) of compound 23

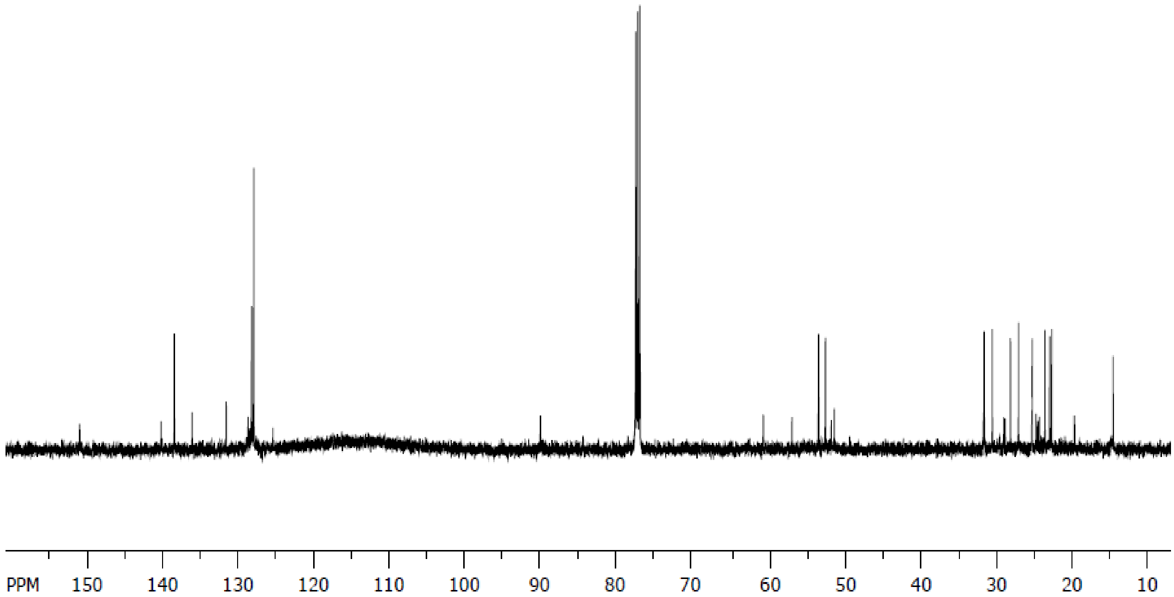

Supplement: File 1 — Experimental procedures and copies of 1H and 13C NMR spectra of compounds 12a, 12b, 13a, 17a, 17b, 18, 19, 20, 21a, 21b, 22a, 22b and 23. [file Beilstein_J_Org_Chem-13-1230-s001.pdf]
